# Microwave-Assisted Protocol for Green Functionalization of Thiophenes With a Pd/β-Cyclodextrin Cross-Linked Nanocatalyst

**DOI:** 10.3389/fchem.2020.00253

**Published:** 2020-04-17

**Authors:** Silvia Tabasso, Emanuela Calcio Gaudino, Elisa Acciardo, Maela Manzoli, Barbara Bonelli, Giancarlo Cravotto

**Affiliations:** ^1^Dipartimento di Chimica, University of Turin, Turin, Italy; ^2^Dipartimento di Scienza e Tecnologia del Farmaco and NIS, Centre for Nanostructured Interfaces and Surfaces, University of Turin, Turin, Italy; ^3^Department of Applied Science and Technology, Politecnico di Torino, Turin, Italy

**Keywords:** microwaves, heterogeneous catalysis, bio-based solvent, C-H arylation, fluorescent quinazolinone, one-pot synthesis

## Abstract

Microwaves (MW) are often the most efficient, in terms of heat exchange and conversion rate, of all the energy sources used to promote chemical reactions thanks to fast volumetric dielectric heating, and metal-catalyzed synthetic reactions under heterogeneous conditions are an eloquent example. We herein report a MW-assisted green protocol for the C-H arylation of thiophenes with substituted aryl halides. This sustainable protocol carried out in γ-valerolactone (GVL) is catalyzed by Pd nanoparticles embedded in cross-linked β-cyclodextrin. In view of the excellent results achieved with activated substrates, the one-pot synthesis of a 4(*3H*)-quinazolinone derivative has been accomplished. A pressure-resistant MW reactor, equipped with multiple gas inlets, was used for sequential (i) C-H arylation, (ii) reduction, and (iii) carbonylation in the presence of the same catalyst, but under different gas atmospheres. The robust heterogeneous Pd catalyst showed limited metal leaching in GVL, making this an efficient MW-assisted process with high atom economy.

**Graphical Abstract:**
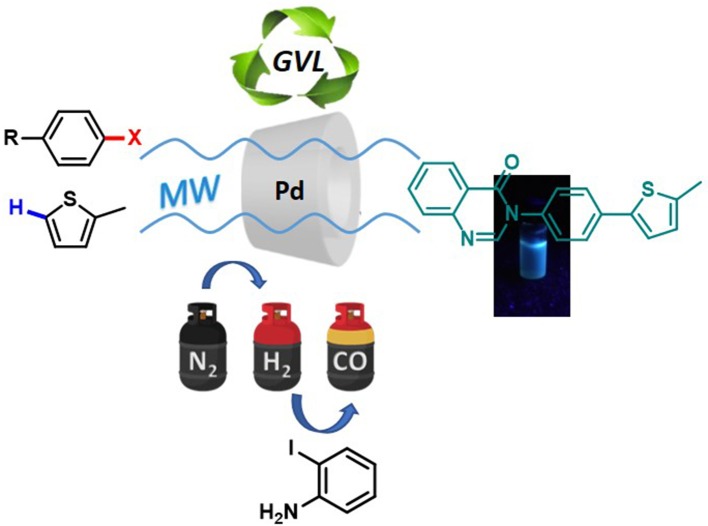
Microwaves-assisted protocol for green functionalization of thiophenes with Pd/β-cyclodextrin cross-linked nanocatalyst.

## Introduction

With the growing concerns of environmental pollution and the depletion of natural resources, synthetic organic chemists are facing the challenge of designing “greener” methodologies (Sheldon, 2012). Effective heating is one of the key means with which to access desired chemical reactivity while ensuring suitable energy consumption. In this context, microwave (MW) dielectric heating is still today one of the most powerful tools for promoting synthetic transformations (De la Hoz and Loupy, 2012; Cravotto and Carnaroglio, 2017; Das et al., 2019).

MW not only significantly shorten reaction times and improve reaction yields and selectivity, but are also more environmentally friendly than traditional heating methods (Kappe, 2004; Moseley and Kappe, 2011). A broad array of new heterogeneous catalytic applications have been reported (Daştan et al., 2012; Cho et al., 2016) since Varma first introduced solid catalysts to MW-assisted organic synthesis (MAOS) (Polshettiwar and Varma, 2008; Polshettiwar et al., 2009). In addition to their recyclability, there are several other advantages to using solid catalysts in MAOS; heterogeneous catalysts are generally excellent MW absorbers, meaning that they provide a cooperative effect in MW-assisted reactions as the catalytic material is also the source of rapid internal heating (Kokel et al., 2017).

The direct functionalization of otherwise inert C–H bonds has recently emerged as a viable alternative (Gandeepan et al., 2019) to traditional cross-coupling reactions, and is a more sustainable approach to transition-metal-catalyzed organic reactions. This approach avoids the pre-functionalization of starting materials and makes C-H activation even more appealing from the viewpoints of high atom and step economy, and high efficiency (Crabtree and Lei, 2017; Meyer et al., 2019).

Despite its many advantages, the development of this concept has also encountered significant challenges; C–H activation processes often require high catalyst loadings (1–10 mol%), (Turner et al., 2007) and phosphine ligands (Masui et al., 2004), and have been performed in toxic dipolar aprotic solvents. Furthermore, achieving regioselective C–H activation is highly challenging as aromatic compounds typically contain multiple C–H bonds. For this reason, the development of new methods for the sustainable synthesis of bi-heteroaryl scaffolds for use as the privileged π-conjugated cores of bioactive compounds, and as functional materials (Ackermann et al., 2009; Gandeepan et al., 2019) is an ongoing challenge for organic chemists. Although ligand-less protocols with low catalyst loadings have recently been developed by Doucet et al., the reaction is still hampered by its harsh conditions and the, up to, 20 h required for completion (Roger et al., 2009).

The use of heterogeneous reusable metal catalysts (Cano et al., 2015; Santoro et al., 2016; Warratz et al., 2017; Orduna et al., 2019), also in combination with MW irradiation technologies (Sharma et al., 2018), is a recent trend in the field of improving the sustainability of C–H activation. However, the quest for less hazardous and more environmentally benign solvents is still of paramount importance for researchers that wish to perform transition-metal-catalyzed C–H activation in an eco-friendly fashion (Fischmeister and Doucet, 2011; Jimenez–Gonzalez et al., 2011; Gutmann et al., 2015; Clarke et al., 2018; Santoro et al., 2018). To this end, alternative approaches for C-H activation have been established, including “on water” (Kitanosono et al., 2018) and “solvent-free” (Martins et al., 2009), reaction conditions. In fact, biomass-derived solvents, such as PEGs, γ-valerolactone (GVL) and 2-methyltetrahydrofuran (MeTHF), have more recently been exploited for transition-metal-catalyzed C–H activation reactions (Santoro et al., 2018). Bio-derived solvents display several advantages, including (i) availability from renewable feedstocks on scale, (ii) generally low toxicities, and (iii) high biodegradability, and may well be a suitable solution to the “solvent issue” in C-H activation processes. In particular, GVL (Rasina et al., 2016), which is typically produced from the hydrogenation of lignocellulosic biomass-derived levulinic acid, has recently been used as a green reaction media for several transition-metal-catalyzed transformations (Tabasso et al., 2016). In particular, Ackermann and Vaccaro reported the first heterogeneous Pd-catalyzed Catellani reaction (Rasina et al., 2016) in GVL, and the same group also exploited the potential of GVL in the heterogeneous Pd-catalyzed direct arylation of 1,2,3-triazoles by aryl bromides (Tian et al., 2016). Although the above-mentioned use of GVL was performed under conventional heating, the interaction between GVL and microwaves has been recently investigated, with the media demonstrating stability and an ability to avoid the arching phenomena that frequently occurs in MW-assisted Pd/C-catalyzed (Petricci et al., 2018) reactions. These results pave the way for its use in MW-assisted heterogeneous catalysis.

In the context of combining MW irradiation and bio-derived reaction media, our laboratory has recently described the direct C–H arylation of thiophenes in biomass-derived GVL under homogeneous catalysis (Tabasso et al., 2017). The ligand-free direct arylation of thiophenes was accomplished in GVL with very low Pd loading, leading to the MW-assisted synthesis of a poly(3-hexyl)thiophene (P3HT), and the role of GVL as a ligand in the catalytic system was also proven.

In the search for more sustainable approaches, we turned toward heterogeneous (Santoro et al., 2016) catalysis for C-H activation. We have recently developed a reusable heterogeneous catalytic system that is based on Pd that has been embedded into a cross-linked β-cyclodextrin (β-CD) matrix (hereafter denoted as Pd/CβCAT system) (Cravotto et al., 2012; Calcio Gaudino et al., 2015, 2017), and obtained after the reticulation of β-CD with hexamethylene diisocyanate (HDI) in the presence of a Pd(II) salt solution (Tabasso et al., 2019). This heterogeneous catalyst has been used, in this work, for the MW-assisted C-H arylation of thiophenes in bio-derived GVL. Furthermore, we propose a one-pot synthesis of a 4(*3H*)-quinazolinone derivative via the reduction and carbonylation of the arylation product of 2-methylthiophene and bromonitrobenzene in GVL. A pressure-resistant MW reactor that is equipped with multiple gas inlets has been used to facilitate the one pot synthesis of 4(*3H*)-quinazolinone in the presence of the same Pd/CβCAT catalyst under different gas atmospheres.

## Experimental Section

All starting organic reagents and solvents were purchased from Sigma Aldrich and used without further purification. β-CD was purchased from Wacker Chemie, Munich, Germany.

The Pd/CβCAT catalyst was prepared according to a previously reported procedure (Tabasso et al., 2019). The cross-linked Pd/CβCAT catalyst was obtained via sonochemical reticulation of β-CD with hexamethylene diisocyanate (HDI) in the presence of a Pd(II) salt solution. In particular, Pd(OAc)_2_ (200 mg, 0.89 mmol) and β-CD (1 g, 0.78 mmol) were dissolved in DMF (4 mL) under sonication in a thermostated sonochemical reactor at room temperature. HDI (2.8 mL, 17.4 mmol) was then added portionwise and the reaction mixture was kept under sonication at 60°C (21.1 kHz, 60 W) for 30 min. The compact gel was crushed and washed with water (100 mL), acetone (100 mL), and methanol (100 mL). The product was filtered on a sintered glass Buchner funnel and dried overnight under vacuum at 75°C, obtaining a brownish powder (4.70 g).

MW-assisted reactions were carried out in a SynthWAVE (MLS GmbH, Milestone Srl) reactor. The GC-MS analyses were performed in an Agilent 6890 system (Agilent Technologies, USA) fitted with a mass detector Agilent Network 5973 with a capillary column that was 30 m long, had an i.d. of 0.25 mm and a film thickness of 0.25 mm. The GC conditions were: injection split of 1:20, injector temperature of 250°C, and detector temperature of 280°C. The gas carrier was helium (1.2 mL min^−1^), and the temperature program ran from 70°C (2 min) to 300°C at 5°C min^−1^.

NMR spectra were recorded on a Jeol 600 ECZ R at 25°C in CDCl_3_; chemical shifts were calculated relative to the residual solvent proton and carbon resonances.

Transmission electron microscopy (TEM) measurements were carried out using a side entry Jeol JEM 3010 (300 kV) microscope, with a LaB_6_ filament, and fitted with X-ray EDS probe via a Link ISIS 200 detector. The samples, in the form of powders, were directly deposited onto a copper grid coated with a porous carbon film. Digital micrographs were acquired using an Ultrascan 1000 camera and processed using Gatan digital micrograph. The images were taken on several different regions of the grid and Pd particle-size distributions were built by counting a statistically representative number of particles (more than 200 particles). The mean particle diameter (d_m_) was calculated as follows: d_m_ = Σd_i_n_i_/Σn_i_, where n_i_ is the number of particles of diameter d_i_. The analyses were firstly performed on the as-synthesized (fresh) catalyst. Other measurements were carried out on used Pd/CβCAT, i.e., after the one-pot 4(*3H*)-quinazolinone synthesis reaction, and on the catalyst submitted to MW power at 200°C.

XPS (X-ray Photoelectron Spectroscopy) analyses were carried out on XPS PHI 5000 Versa probe apparatus using a band-pass energy of 187.85 eV, a 45° take-off angle and a 100.0 μm diameter X-ray spot size for survey spectra. High-resolution XP spectra were recorded under the following conditions: pass energy of 20 eV, resolution of 0.1 eV and step of 0.2 eV. Sample charging effects were eliminated by referring to the spectral line shift of the C 1 s binding energy (BE) value at 284.5 eV. XP-spectra were analysed using commercial software (CasaXPS, version 2.3.16), and applying mixed Gaussian-Lorentzian (70–30%) profiles.

### General Procedure for Direct Arylation of Thiophenes

The aryl halide (0.5 mmol), thiophene derivative (1 mmol), KOAc (1 mmol), and PivOH (0.15 mmol) were placed into a quartz vial equipped with a magnetic stirrer and suspended in GVL (3 mL). The vial was purged with N_2_ and Pd/CβCAT (0.2 mol%) was added. The mixture was heated to the required temperature under MW irradiation for 2 h in a N_2_ (1 MPa) atmosphere under magnetic stirring (450 rpm). The reactor was cooled after 2 h and the crude reaction was filtered to recover the catalyst. Water (4 mL) was added to the filtrate and the solid products were recovered via precipitation, filtered off and washed with 1 mL of cold water. The solid was then dried under vacuum and purified by flash column chromatography (hexane). Alternatively, liquid products were extracted using 3 mL EtOAc and purified by flash column chromatography (hexane) after solvent evaporation.

### One-Pot Synthesis of 4(*3H*) Quinazolinone Derivative

The aryl bromide **2a** (0.5 mmol), 2-methylthiophene **1a** (1 mmol), KOAc (1 mmol) and PivOH (0.15 mmol) were placed into a quartz vial equipped with a magnetic stirrer and suspended in GVL (3 mL). The vial was purged with N_2_ and Pd/CβCAT (0.2 mol%) was added. The mixture was heated to 140°C under MW irradiation (average power 960 W) in a N_2_ (1 MPa) atmosphere under magnetic stirring (450 rpm). After 2 h, H_2_ (15 bar) was flushed into the reaction mixture for 15 min, and the temperature was increased to 160°C. The residual internal pressure was carefully released after the reaction chamber was cooled to 35°C. O-iodoaniline (0.5 mmol) and trimethyl orthoformate (0.6 mmol) were added and CO pressure (5 bar) was loaded at room temperature. The mixture was heated at 145°C for 4 h under magnetic stirring. After the reaction chamber was cooled, the Pd catalyst was filtered off on a sintered-glass Buchner funnel and washed twice with GVL. Water (3 mL) was then added to the filtrate solution, and the product was recovered after precipitation, filtered off and washed with 1 mL of cold water. The obtained precipitate was filtered off, washed twice with water, and finally dried under vacuum. Product purification was performed via flash-chromatography (petroleum ether 40–60/EtOAc = 7:3 v/v), yielding the 4(*3H*)-quinazolinone derivative (6).

## Results and Discussion

### Direct Arylation of Thiophenes

The polar nature of cyclodextrins makes them good candidates for interaction with MW, meaning that they are therefore particularly efficient at adsorbing the irradiation. For this reason, they can be used as supports for metals (Hapiot et al., 2014) in MW-assisted reactions (Cintas et al., 2012; Cravotto et al., 2012). In addition, the structural features of aromatic compounds mean that they fit the cyclodextrin cavity well (Connors, 1997).

**Scheme 1:**
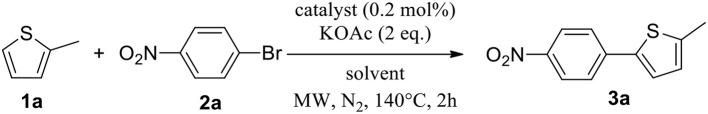
C-H direct arylation of 2-methythiophene with 4-bromonitrobenezene.

In a previous work, we described the ligand free C-H arylation of thiophenes in GVL (Tabasso et al., 2017) using Pd(OAc)_2_ as the catalyst and potassium acetate (KOAc) as the base. Herein, we explore the same reaction, under the previously optimized conditions, but using a heterogeneous Pd/cross-linked β-CD catalyst (Pd/CβCAT), both to improve the sustainability of the process and to investigate the active catalytic species that are formed under the MW-assisted reaction. The direct C-H arylation between 2-methylthiophene and 4-bromonitrobenzene (Scheme 1) was investigated, as the first phase, and the Pd/cross-linked β-CD catalyst was compared to Pd(OAc)_2_ (Table 1). The metal content in the homogeneous and heterogeneous catalysis reactions was the same (0.2 mol%). The green solvent, GVL, was also compared to dimethylacetamide (DMAc), one of the most commonly used conventional dipolar aprotic solvents.

**Table 1:** MW-assisted C-H direct arylation of 2-methythiophene with 4-bromonitrobenezene.

**Entry**	**Ligand**	**Catalyst**	**Solvent**	**Yield^a^ (%)**
1	–	Pd(OAc)_2_	DMAc	100
2	–	Pd/CβCAT	DMAc	100
3	–	Pd(OAc)_2_	GVL	73
4		Pd/CβCAT	GVL	78 (81)^b^
5^c^	–	Pd/CβCAT	GVL	51
6^d^	–	Pd/CβCAT	GVL	42
7^e^	–	Pd/CβCAT	GVL	36
7	PivOH (0.3 eq.)	Pd(OAc)_2_	GVL	82
8	PivOH (0.3 eq.)	Pd/CβCAT	GVL	100

a *Yields as determined by GC*;

b *140°C, 4 h*;

c 160°C, 2 h

d *180°C, 2 h*;

e *200°C, 2 h*.

Pd/CβCAT showed itself to be a valuable alternative to homogeneous catalysis when used in conventional solvents, as shown in Table 1, entry 2. However, the product yield was lower in GVL, as reagent conversion did not complete, even when the reaction time was prolonged to 4 h (Table 1, entry 4b) and when the temperature was raised (Table 1, entry 5, 6, and 7). As described more in detail in Paragraph 2.3 and in the Supplementary Material, the increase of MW power at temperatures higher than 140°C causes a higher MW adsorption by cross-linked CD structures, that negatively affect its stability, as confirmed by TEM analysis (Figures 3b,c). Aiming to promote the reaction, we added PivOH as a ligand (in the form of pivalate, Lafrance and Fagnou, 2006). This improvement resulted in complete conversion to the product (Table 1, entry 8). It is worth noting that the heterogeneous catalyst was more efficient than Pd(OAc)_2_ when used in GVL. This may be due to the higher stability of the active Pd species in the catalyst network, thus avoiding the formation of Pd black, as will be discussed below (section Effect of Microwaves on the Pd/β-CD Cross-Linked Nanocatalyst). Indeed, as Pd(0) is the catalytically active species, metal aggregation (and catalyst deactivation) can occur when using Pd(OAc)_2_, as GVL is less efficient than DMAc in coordinating Pd atoms (Tabasso et al., 2017). The optimized conditions were then applied to other substrates and the action of the two catalysts Pd(OAc)_2_ and Pd/CβCAT was compared (Table 2).

**Table 2:** MW-assisted coupling of heteroaromatics and aryl halides.

**Entry**	**Heteroaryl**	**Aryl halide**	**Product**	**Yield (%)^a^ Pd(OAc)_2_**	**Yield (%)^a^ Pd/CβCAT**
1	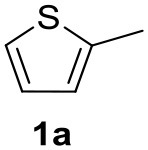	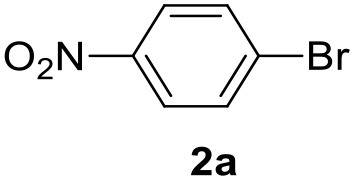	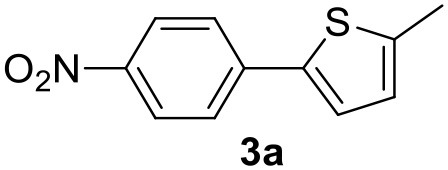	82	99
2	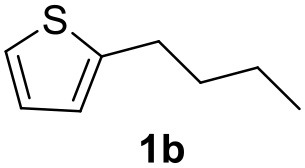	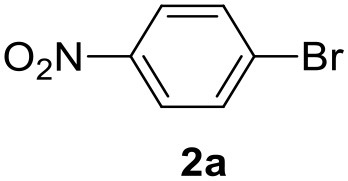	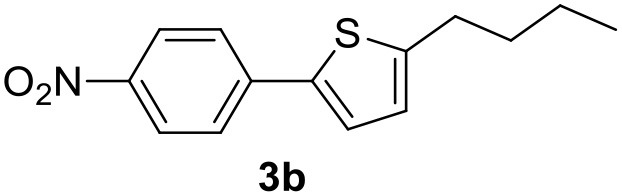	76	98
3	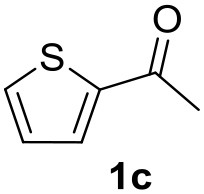	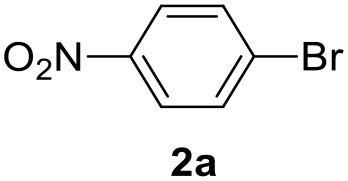	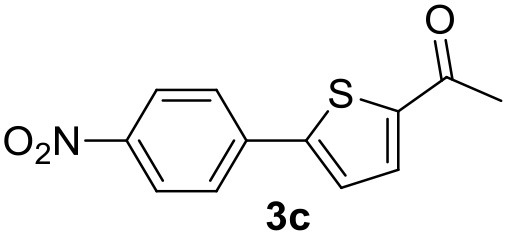	28	84
4	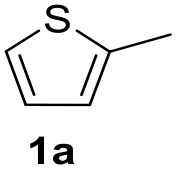	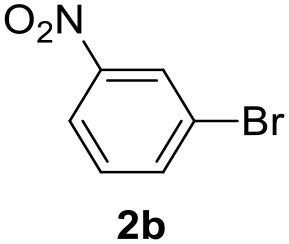	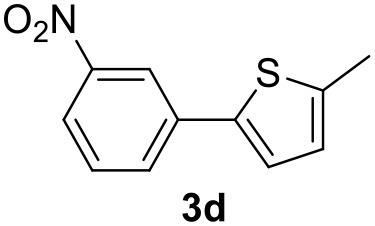	80	90
5	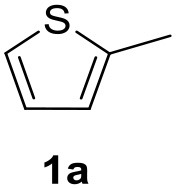	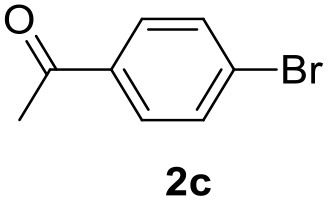	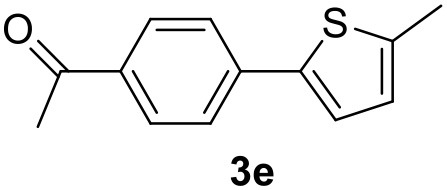	66	62
6	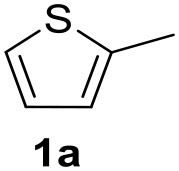	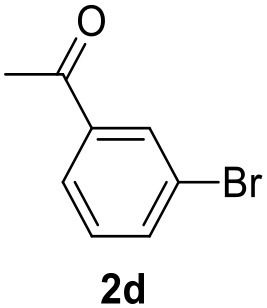	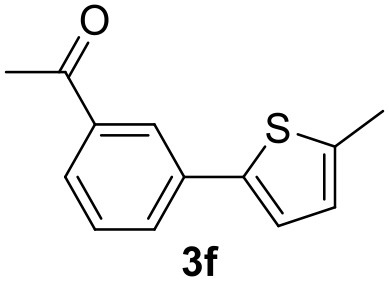	13	54
7	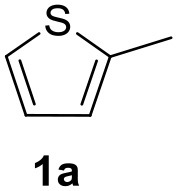	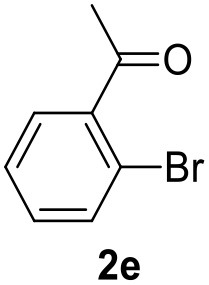	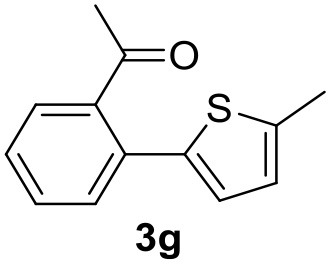	0	0
8	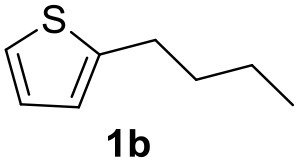	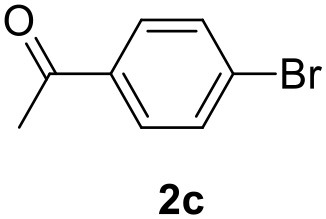	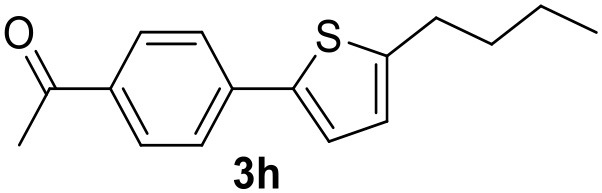	28	0
9	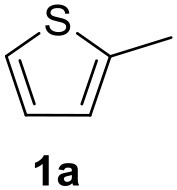	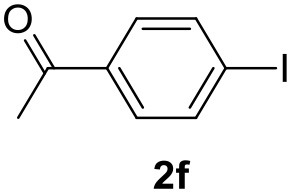	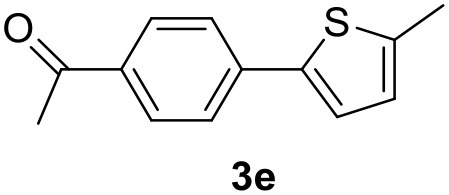	14	10
10^b^	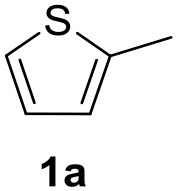	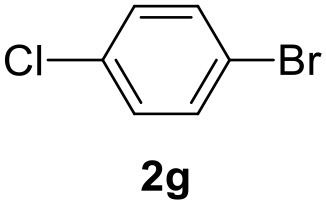	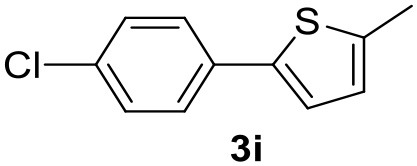	0	0
11	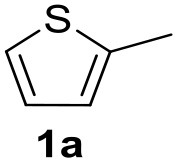	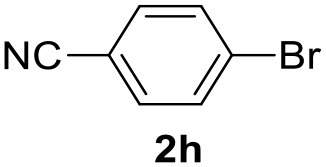	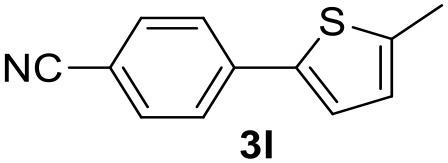	90	93
12	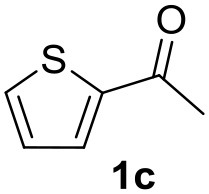	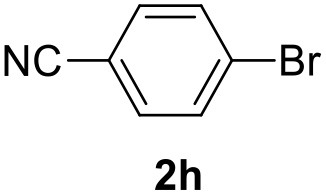	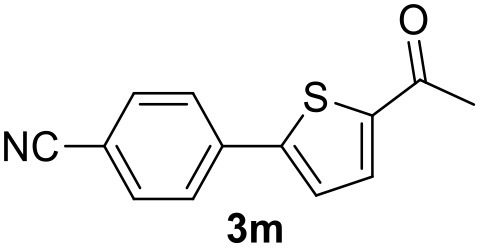	86	74
13	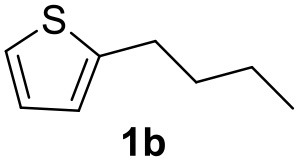	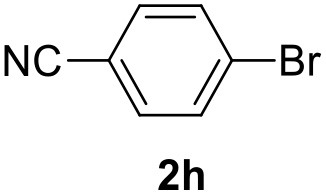	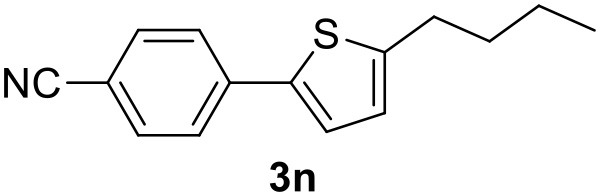	96	73
14	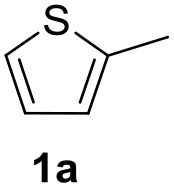	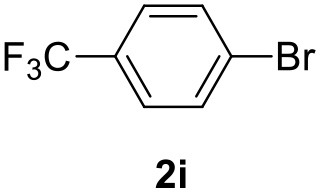	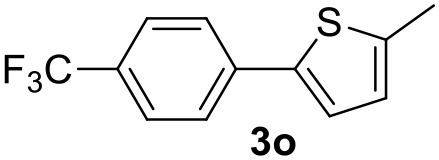	35	41
15	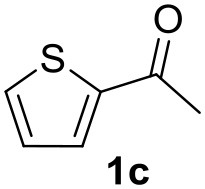	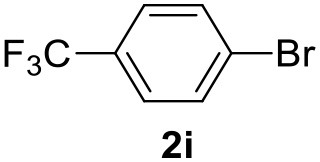	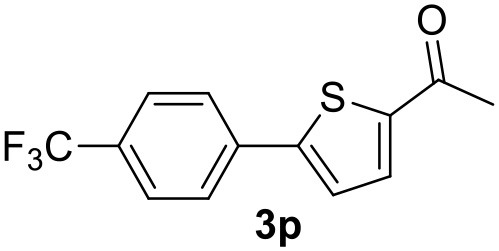	56	60
16	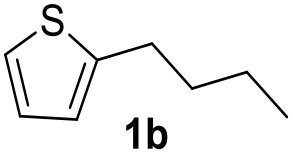	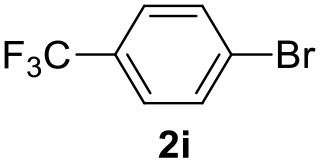	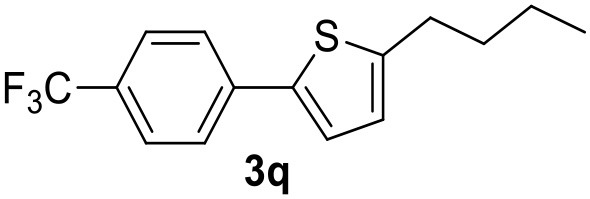	48	32
17	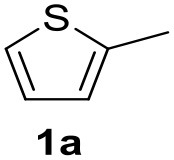	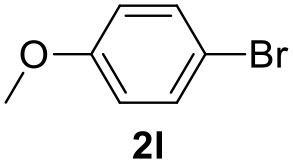	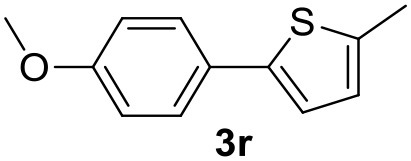	0	0
18	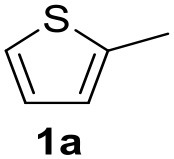	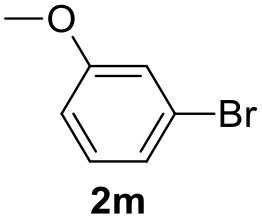	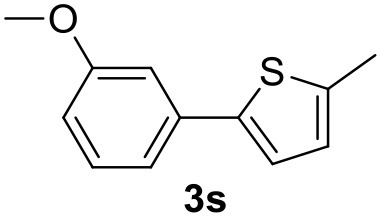	0	0
19	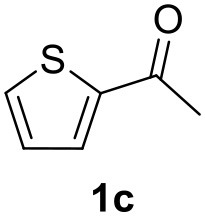	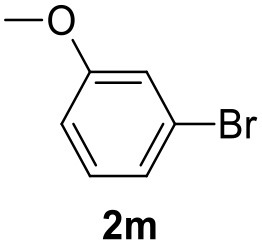	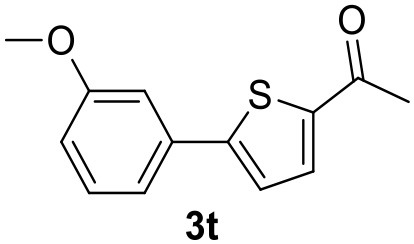	0	0
20	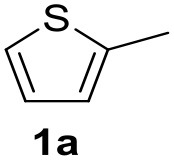	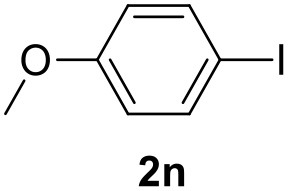	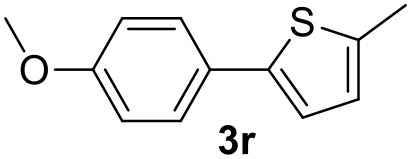	42	24
21	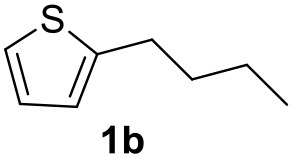	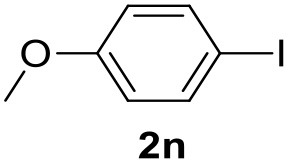	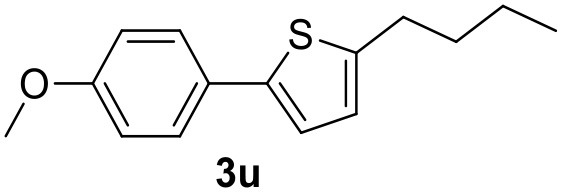	50	44
22	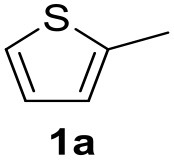	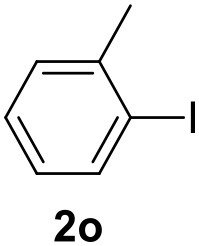	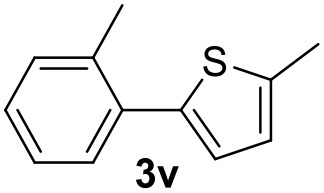	52	19
23^c^	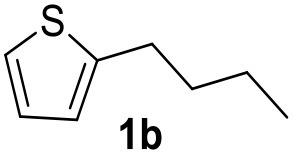	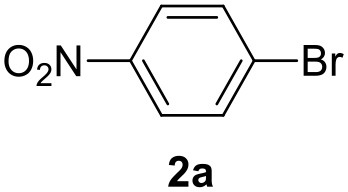	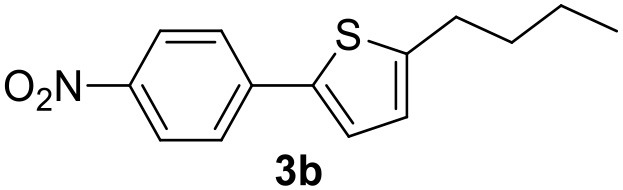	2	3

*Reaction conditions: the mixture of aryl halide (0.5 mmol), thiophene derivatives (1 mmol), KOAc (1 mmol) PivOH (0.15 mmol), Pd(OAc)_2_ or Pd/CβCAT (0.2 mol%). and GVL (3 mL) was heated to 140°C under MW irradiation for 2 h*.

a *Yields as determined by GC*.

b *Reaction performed at 200°C*.

c *Conventional heating*.

It was observed that MW significantly promoted the reaction, and the same reaction gave much lower yields under conventional heating (Table 2, entry 23 and Table S1). In particular, the complete conversion of the substrate took longer without MW irradiation; the compounds detected in the reaction mixture were mainly the reagents. Electron-deficient aryl halides, such as 1-bromo-4-nitrobenzene and 1-bromo-3-nitro benzene, react smoothly with thiophenes (Table 2, entries 1–4). Quite surprisingly, the heterogeneous catalyst had a much stronger effect on the reaction of 2-acetylthiophene (Table 2, entry 3) than Pd(OAc)_2_. This may be due to the cyclodextrins being highly responsive to MW irradiation, leading to the rapid heating of the catalytic site. In some cases, the higher yields obtained using Pd/CβCAT can be ascribed to the tridimensional substrate structures having a good fit within the catalyst framework, such as in the coupling of 3-bromoacetophenone and methylthiophene (Table 2, entry 6). On the other hand, heterogeneous reaction yields were often lower than the homogeneous reactions when using n-butylthiophene as a reagent (Table 2, entries 8, 13, 16, 21). These results may be caused by the n-alkyl moiety's strong binding to cyclodextrins (expressed by pKd = –log K), which increases monotonically with alkyl chain length, up to about eight carbons (Tee et al., 1996); the higher stability of the *n*-butylthiophene-cyclodextrin complex means that the reaction proceeds more slowly.

The attempt to couple deactivated aryl halides and thiophene was quite disappointing as only aryl iodides led to the desired products in the presence of the methoxy group (Table 2, entries 20, 21, 22), and the homogeneous catalyst gave higher yields.

The reusability of the catalyst was investigated by repeating the optimized reaction procedure between 2-methythiophene and 4-bromonitrobenezene with a used catalyst, after washing it with water, methanol and acetone. After the first Pd/CβCAT recycle the product yield remains high (90%) with a negligible metal leaching (0.98%) as confirmed by ICP-OES. However, the comparison between the XRD patterns of the as synthesized and recycled catalyst (Figure S4) revealed the formation of some peaks possibly ascribed to the PdS_2_ crystal phase (JCPDS file number 00-011-0497) along with the presence of peaks related to cubic Pd (JCPDS file number 00-001-1201). The broad peaks related to β-CD crosslinked with HDI moieties do not significantly change after recycling. However, the formation of the S-containing Pd species can poison the catalyst.

### Synthesis of a 4(*3H*)-Quinazolinone Derivative

These encouraging results, especially with activated substrates, drove us to apply this process to the synthesis of a 4(*3H*)-quinazolinone derivative. These compounds have been widely explored because of the broad scope of their beneficial biological activities (Cao et al., 2005; Khan et al., 2016). A great deal of effort has been invested in the development of easy-to-handle, cost-effective and eco-friendly protocols for their preparation. An environmentally sustainable synthesis for 4(*3H*)-quinazolinone moieties that proceeds via MW-assisted carbonylative reactions in GVL has recently been published (Calcio Gaudino et al., 2017).

**Scheme 2:**
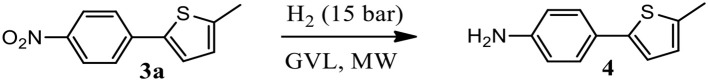
Optimized conditions for the MW-assisted reduction step.

The first step of this process, the MW-assisted reduction of 2-methyl-5-(4-nitrophenyl)thiophene (Scheme 2), was therefore optimized in GVL. However, the product yield was not satisfactory (25%) when working with 0.2% of Pd. We therefore decided to use a higher amount of catalyst (10% mol Pd), as has recently been described for reduction reactions under MW dielectric heating, and product **4** was obtained in a quantitative yield in only 15 min at 120°C.

**Scheme 3:**
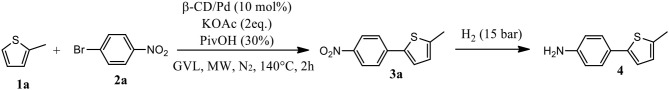
Sequential direct arylation and reduction reactions.

However, it was necessary to increase the temperature to 160°C to achieve complete conversion to desired product **4** (Scheme 3) when performing the one-pot reaction between 2-methythiophene and 4-nitrobenzene (Table 3, entry 2). Reaction times could not be reduced any further with this temperature increase (Table 3, entry 3).

**Table 3:** Optimization of the second step of the one-pot synthesis of 2-methyl-5-(4-aminophenyl)thiophene.

**Entry**	**T (^°^C)**	**Time**	**Yield (%)^a^**
1	120	15′	9
2	160	15′	100
3	160	5′	11

a *Yields as determined by GC*.

**Scheme 4:**
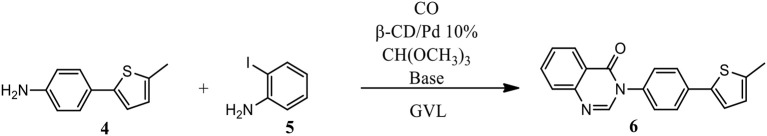
Carbonylative coupling reaction.

The synthesis of the 4(*3H*)-quinazolinone derivative was then accomplished via a carbonylative coupling of 2-methyl-5-(4-aminophenyl)thiophene with *o*-iodoaniline, according to a previously reported procedure (Calcio Gaudino et al., 2017), using trimethyl orthoformate under CO pressure (Scheme 4). We have compared the previously used base, triethylamine (TEA), with KOAc, which is the base used in the C-H activation step. As shown in Table 4, increasing the time, temperature and CO pressure led to higher product yields being achieved using KOAc (entry 3) than with TEA (entry 4).

**Table 4:** Optimization of the conditions for the carbonylative coupling reaction.

**Entry**	**Base**	**CO (bar)**	**T (^°^C)**	**Time (h)**	**Yield^a^ (%)**
1	KOAc	2.5	125	1	–
2	TEA	7.5	125	3	30
3	KOAc	5	145	4	88
4	TEA	5	145	4	60

a *Yields as determined by GC*.

**Scheme 5:**
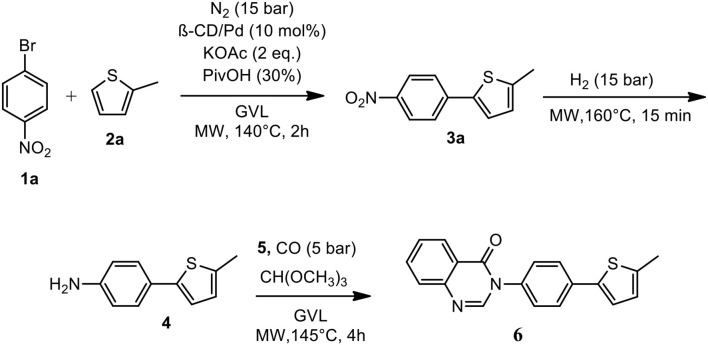
One-pot synthesis of the 4(*3H*)-quinazolinone derivative.

The optimized conditions for each step were applied to a one-pot synthesis, which was made possible by the multiple gas inlets of the MW reactor that allow different reactive gas atmospheres to be used in sequence. Sequential C-H arylation, reduction and carbonylative coupling reactions were therefore performed in GVL using the same heterogeneous catalyst, and KOAc as the base (Scheme 5). Although higher Pd content (10% mol) was used for the synthesis of the 4(*3H*)-quinazolinone derivative, metal leaching, as determined by ICP-OES, was observed to be very low after the one-pot reaction (1.04%). This is due to both the polymer network of the catalyst and the solvent, as GVL is known to be an optimal medium for limiting Pd leaching.

**Figure 1:**
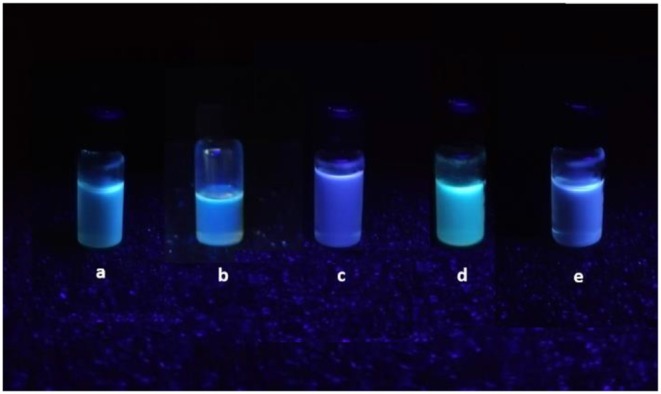
Quinazolinone **6** fluorescence**: (a)** CH_3_CN, **(b)** CH_2_Cl_2_, **(c)** EtOAc, **(d)** MeOH, **(e)** acetone.

The newly synthesized quinazolinone derivative **6** showed interesting fluorescence properties and a solvatochromic effect when illuminated with ultraviolet light (Figure 1).

4(*3H*)-quinazolinone derivatives are not so well-known for their luminescent properties, whereas thiophene-based materials have undergone extensive investigations into their emissive applications (Rasmussen et al., 2015). Nevertheless, the fluorescence properties of potentially bioactive compounds, for example, antiviral, antimalarial and anticancer agents, may potentially permit the highly useful *in-vitro* monitoring of compound-treated cultured cells by fluorescence microscopy. 4(*3H*)-quinazolinone derivative **6** showed promising fluorescence intensity when excited at 300 nm (Figures S1a,b).

### Effect of Microwaves on the Pd/β-CD Cross-Linked Nanocatalyst

TEM combined with EDS measurements and XPS analyses were carried out on the as-synthesized (fresh) and used, i.e., after the one-pot 4(*3H*)-quinazolinone synthesis reaction, catalyst to investigate the effect of MW on the morphology, Pd dispersion and nature of the species exposed on the surface of Pd/CβCAT. It is worth noting that both fresh and used Pd/CβCAT catalysts were found to be stable to prolonged exposure to the electron beam of the instrument, in terms of Pd nanoparticle size and morphology (no metal coalescence), during the TEM measurements. Analogously, the cross-linked β-cyclodextrin support also showed no modifications, proving the intrinsic stability of the framework.

**Figure 2:**
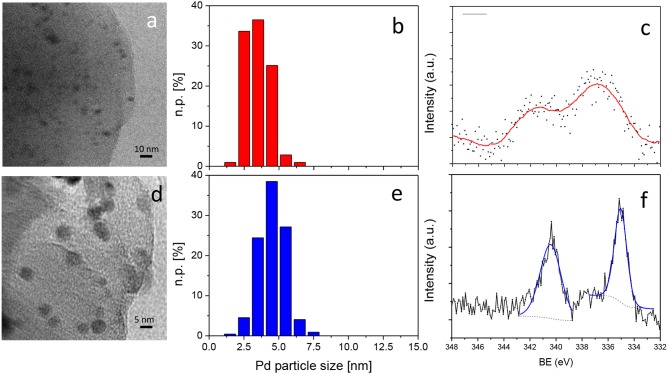
TEM representative images of **(a)** fresh Pd/CβCAT and **(d)** Pd/CβCAT after the one-pot 4(*3H*)-quinazolinone synthesis reaction. Corresponding Pd particle-size distributions (**b,e**, respectively). Instrumental magnification: 150,000 × and 300,000 ×, respectively. n.p. [%] = number of counted particles of diameter d_i_. XP-spectra of the fresh **(c)** and used **(f)** samples in the Pd 3d line range. Section **(c)** the red curve is obtained from the interpolation of the XPS signal. Section **(f)** the blue curve is the result of the curve-fitting procedure and the dotted line is the background.

Pd nanoparticles with a roundish shape were observed on the fresh catalyst (Figure 2a). A significant fraction of these had sizes between 2 and 4 nm, giving a mean diameter of 3.4 ± 0.8 nm (Figure 2b), which highlights the stabilization of the nanoparticles by the support. Moreover, XPS analyses revealed that the surface atomic percentages of Pd (at. % Pd) were 0.3 and 0.2, in the fresh and used samples, respectively, which correspond to ca. 2.3 (fresh sample) and 1.6 (used sample) wt. % Pd. This demonstrates that some leaching occurred during the MW-assisted reaction, as revealed by ICP.

The XP spectra in the binding-energy (BE) range of the Pd 3d line are reported in Figure 2. The fresh-sample spectrum (Figure 2c), in particular, was extremely noisy and no reliable curve-fitting procedure could be performed; a broad, weak signal (dots) was observed, and the red line is an interpolated curve with two maxima at ca. 396.9 and 341.4 eV, i.e., at BE values that are in agreement with the presence of Pd^2+^ species, as is expected considering the synthesis procedure adopted. The XP spectrum, recorded after Ar sputtering (not reported), with the aim of improving the signal-to-noise ratio, was not reliable, as the sputtering procedure probably modified the sample surface. Indeed, XPS analyses of Pd-containing samples is often complicated by PdO reacting with X-rays, when it is present, and the fact that Pd nanoparticles, when present, are often quickly oxidized upon the sample's exposure to air, usually forming core-shell Pd-PdO nanoparticles (Voogt et al., 1996).

The Pd nanoparticles showed contained agglomeration upon the MW-assisted process; a mean diameter of 4.5 ± 1.2 nm was estimated and the particle-size distribution appeared to be shifted toward sizes of 4–5 nm (Figure 2e).

Both the EDS analyses and XPS revealed the presence of S (1.31 and 0.95 wt. %, respectively) at the surface of used Pd/CβCAT (see also Figure S3). This is most likely due to the adsorption of some of the reaction products (seeing as complete conversion was observed during the reaction, the presence of unreacted species can be reasonably ruled out) that were not removed by washing.

Differently from what obtained for the fresh catalyst, the XP spectrum of the used sample, in the Pd 3d range, (Figure 2f) was curve-fitted with a single component, with maxima at 335.05 eV (Pd 3d 5/2) and 340.45 eV (Pd 3d 3/2), with a splitting of 5.40 eV. The observed shift and splitting are in agreement with literature values reported for Pd^0^, as the Pd 3d 5/2 line of bulk Pd^0^ is found at 335.1 eV, with a separation of *ca*. + 5.3 eV from the Pd 3d 3/2 (Rao et al., 2017).

**Figure 3:**
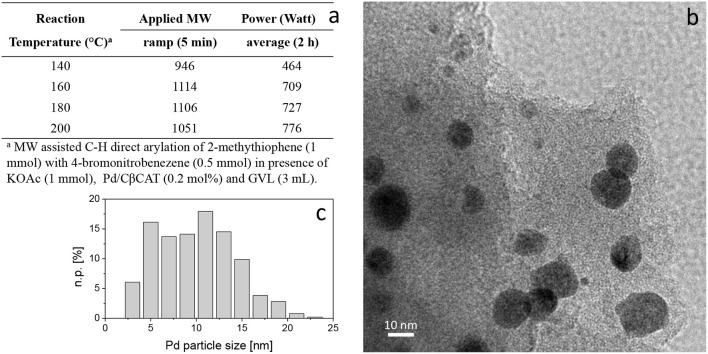
MW-assisted C-H direct arylation: influence of MW power on Pd/CβCAT stability **(a)**. TEM representative image of Pd/CβCAT under average power of 776 W at 200°C **(b)**, and corresponding Pd particle-size distribution **(c)**. Instrumental magnification: 150000 ×, n.p. [%] = number of counted particles of diameter d_i_.

Aiming to investigate the influence of MW power on the stability of metallic nanoparticles and catalytic activity, tests at higher temperatures (160, 180, and 200°C, Table 1, entries 5, 6 and 7) were performed. Figure 3a reports the applied MW power (5 min ramp and 2 h average power) on a reaction carried out at different temperatures. Moreover, the power variation with temperature is reported in Graphs S1a–d.

Due to the MW adsorption by the cross-linked β-CD framework, further agglomeration of the Pd nanoparticles occurred when increasing the MW power at 200°C, as shown in Figure 3b. Indeed, the mean diameter of the Pd nanoparticles increased up to 10.0 ± 4.1 nm (Figure 3c), which negatively affected the Pd/CβCAT stability. In addition, on the stressed catalyst the presence of peaks related to residual unreacted adsorbed species was detected by EDS analysis (Figure S5).

## Conclusions

In this work, we have developed a sustainable protocol for the direct arylation of thiophenes using a heterogeneous Pd catalyst and a green solvent, GVL, which is suited for use in MW-assisted reactions. This process takes advantage of the cooperative effect between MWs and Pd/CβCAT, since the reaction does not proceed at all under traditional heating using the same catalyst and conditions. MW irradiation led to the formation of catalytically active Pd nanoparticles that are stabilized by the polymeric network. These findings were then applied to an atom-economical methodology that yielded a fluorescent quinazolinone derivative. The developed protocol is simple and cheap, avoids tedious workup procedures and works efficiently under MW irradiation. The robust heterogeneous Pd catalyst showed limited metal leaching (1.04%) in GVL. The generation of bioactive heterocycles in a multistep one-pot process avoids the need to isolate intermediates and perform purification steps making it unbeatable in terms of costs and waste reduction, and thus an efficient and sustainable process.

## Data Availability Statement

All datasets generated for this study are included in the article/Supplementary Material.

## Author Contributions

ST and EC: methodology experimental design. EA, ST, and EC: investigation. ST and MM: data curation. MM and BB: catalyst analysis. ST and EC: original draft preparation. GC: review and editing.

## Conflict of Interest

The authors declare that the research was conducted in the absence of any commercial or financial relationships that could be construed as a potential conflict of interest.
