# Supplementary material for: Microwave-Assisted Protocol for Green Functionalization of Thiophenes With a Pd/β-Cyclodextrin Cross-Linked Nanocatalyst
Source: Front Chem. 2020 Apr 17;8:253. doi: 10.3389/fchem.2020.00253 (PMC7180232; doi:10.3389/fchem.2020.00253)

Supplementary Material

**Supplementary data**

**Graph S-1**. MW-assisted C-H direct arylation of 2-methythiophene with 4-bromonitrobenezene: Graphs of power output at different reaction temperatures.


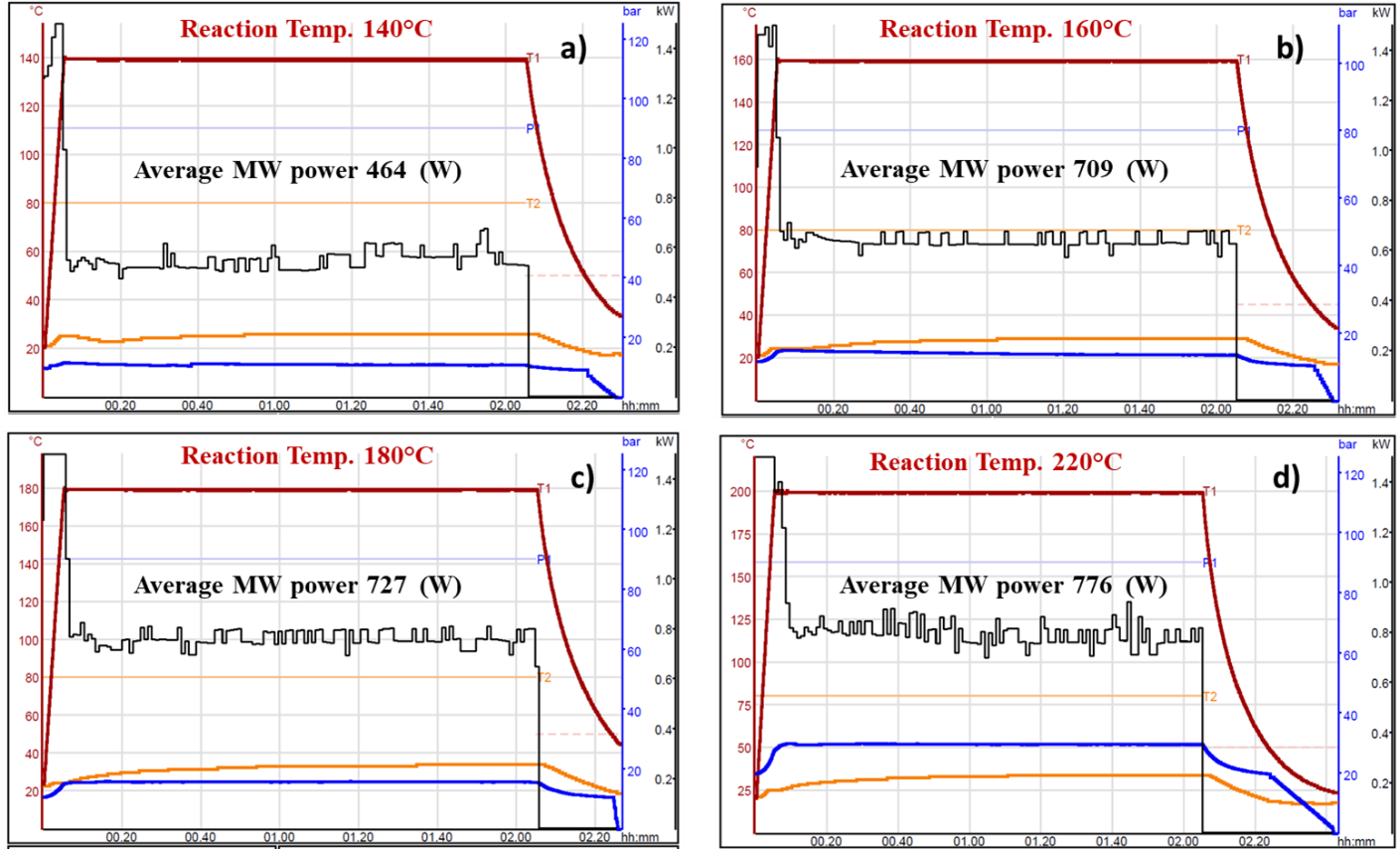


##### MW-assisted C-H direct arylation of 2-methythiophene (1 mmol) with 4-bromonitrobenezene (0.5 mmol) in presence of KOAc (1 mmol), Pd/CβCAT (0.2 mol%) and GVL (3 mL) performed at a) 140°C, b) 160°C, c) 180°C, d) 200°C.

##### Table S-1. MW *vs* Conventional heating for coupling of heteroaromatics and aryl halides

| **Entry** | **Heteroaryl** | **Aryl halide** | **Product** | **Yield (%)^a^**  **Pd(OAc)_2_** | **Yield (%)^a^**  **Pd/CβCAT** |
| --- | --- | --- | --- | --- | --- |
| **1** |  |  |  | 82 (2)^b^ | 99 (3)^b^ |
| **2** |  |  |  | 76 (2) ^b^ | 98 (4) ^b^ |
| **3** |  |  |  | 28 (0) ^b^ | 84 (3) ^b^ |
| **4** |  |  |  | 80 (2) ^b^ | 90 (4) ^b^ |
| **5** |  |  |  | 66 (traces) ^b^ | 62 (traces) ^b^ |
| **11** |  |  |  | 90 (3) ^b^ | 93 (5) ^b^ |
| **12** |  |  |  | 86 (2) ^b^ | 74 (3) ^b^ |
| **13** |  |  |  | 96 (3) ^b^ | 73 (traces) ^b^ |

Reaction conditions: the mixture of aryl halide (0.5 mmol), thiophene derivatives (1 mmol), KOAc (1 mmol) PivOH (0.15 mmol), Pd(OAc)2 or Pd/CβCAT (0.2 mol%). and GVL (3 mL) was heated to 140°C under MW irradiation for 2 hours. ^a^Yields as determined by GC; ^b^ Conventional heating.

# Supplementary Figures

**Figure S-1. Spectroscopic properties** of 3-(4-(5-methylthiophen-2-yl)phenyl)quinazolin-4(3H)-one (**6**): assorbance (a) and fluorescence (b) spectra.


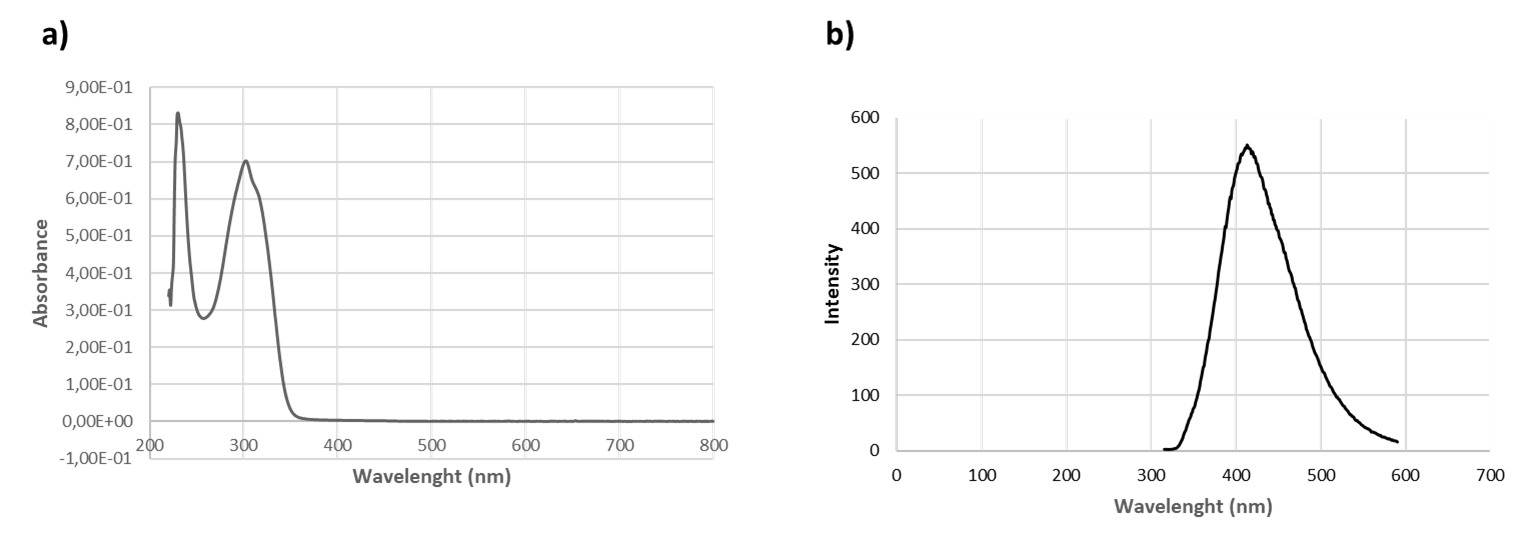


**Figure S-2.** EDS spectrum of the fresh Pd/CβCAT catalyst.

**Figure S-3.** EDS spectrum of the Pd/CβCAT catalyst after the one pot 4(3H)-quinazolinone synthesis reaction.

**Figure S-4.** XRD patterns of the as synthesized (red) and recycled (blue) Pd/CβCAT catalyst.

**Figure S-5.** EDS spectrum of the Pd/CβCAT catalyst after the MW-assisted C-H direct arylation of 2-methythiophene with 4-bromonitrobenezene reaction under MW irradiation at 200 °C.

# NMR data

**2-methyl-5-(4-nitrophenyl)thiophene (3a)**

**^1^H NMR** (600 MHz, CDCl_3_): δ 8.20 (d, J = 9.0 Hz, 2H), 7.66 (d, J = 9.0 Hz, 2H), 7.28 (d, J = 3.6 Hz, 1H), 6.78 – 6.81 (m, 1H), 2.53 (s, 3H) ppm; **^13^C NMR** (150 MHz, CDCl_3_): δ 146.3, 143.1, 141.0, 139.2, 127.2, 125.9, 125.5, 124.5, 15.8 ppm.


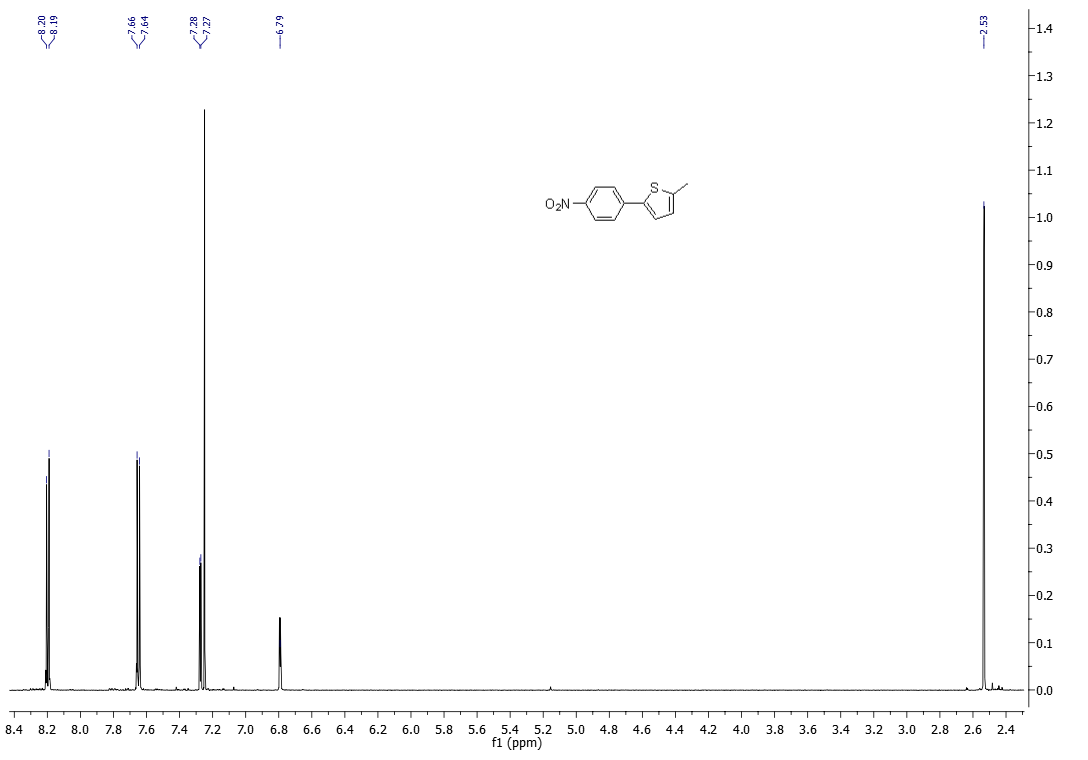


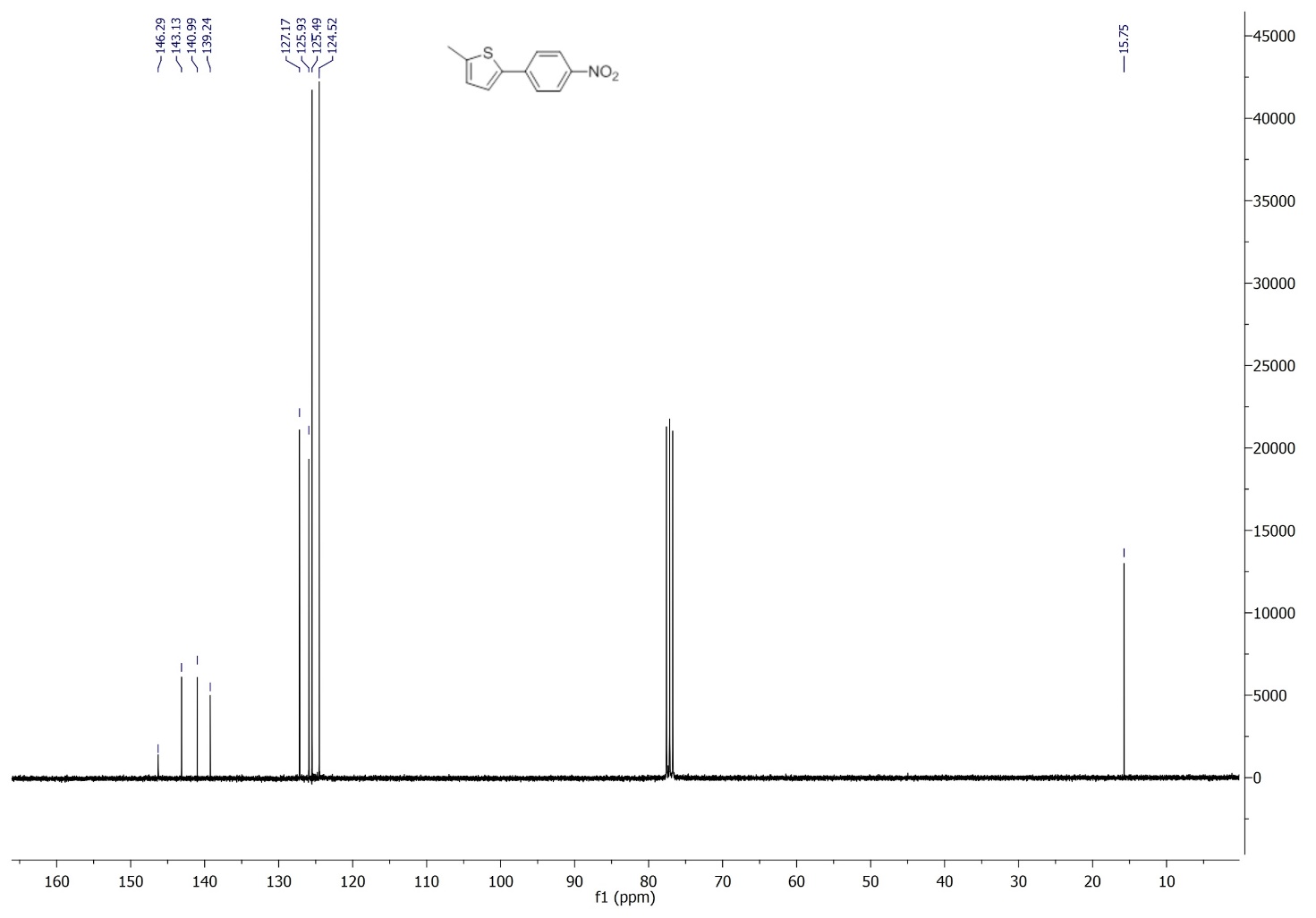


**2-butyl-5-(4-nitrophenyl)thiophene (3b)**

**^1^H NMR** (600 MHz, CDCl_3_): δ 8.13 (d, *J* = 8.6 Hz, 2H), 7.59 (d, *J* = 8.6 Hz, 2H), 7.22 (d, *J* = 3.3 Hz, 1H), 6.74 (d, *J* = 2.5 Hz, 1H), 2.78 (t, *J* = 7.5 Hz, 2H), 1.65 – 1.60 (m, 2H), 1.39 – 1.31 (m, 2H), 0.88 (t, *J* = 7.3 Hz, 3H) ppm; **^13^C NMR** (150 MHz, CDCl_3_): δ 149.3, 146.3, 141.1, 138.9, 126.0, 125.7, 125.5, 124.5, 33.8, 30.2, 22.3, 13.9 ppm.

**
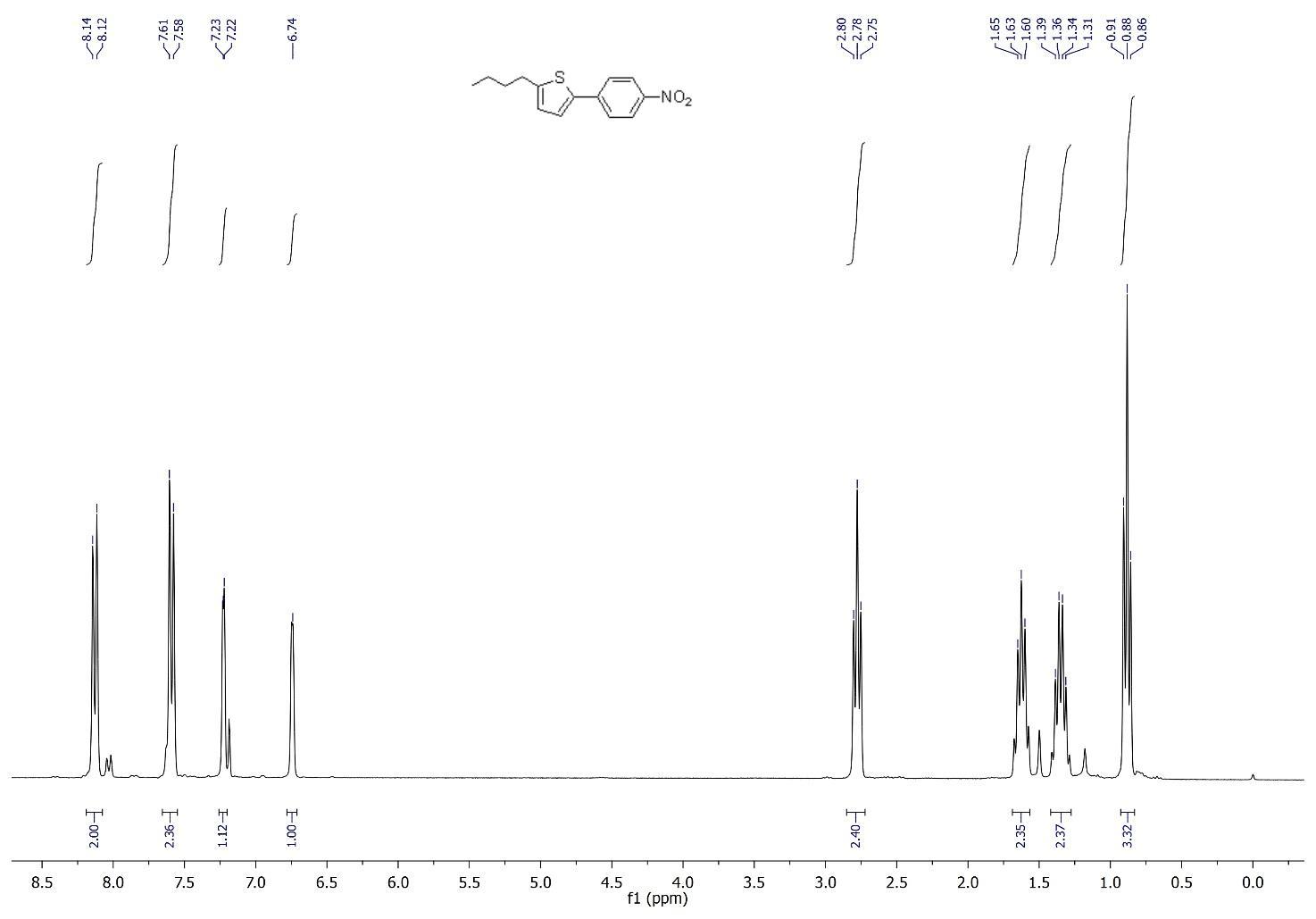
**

**
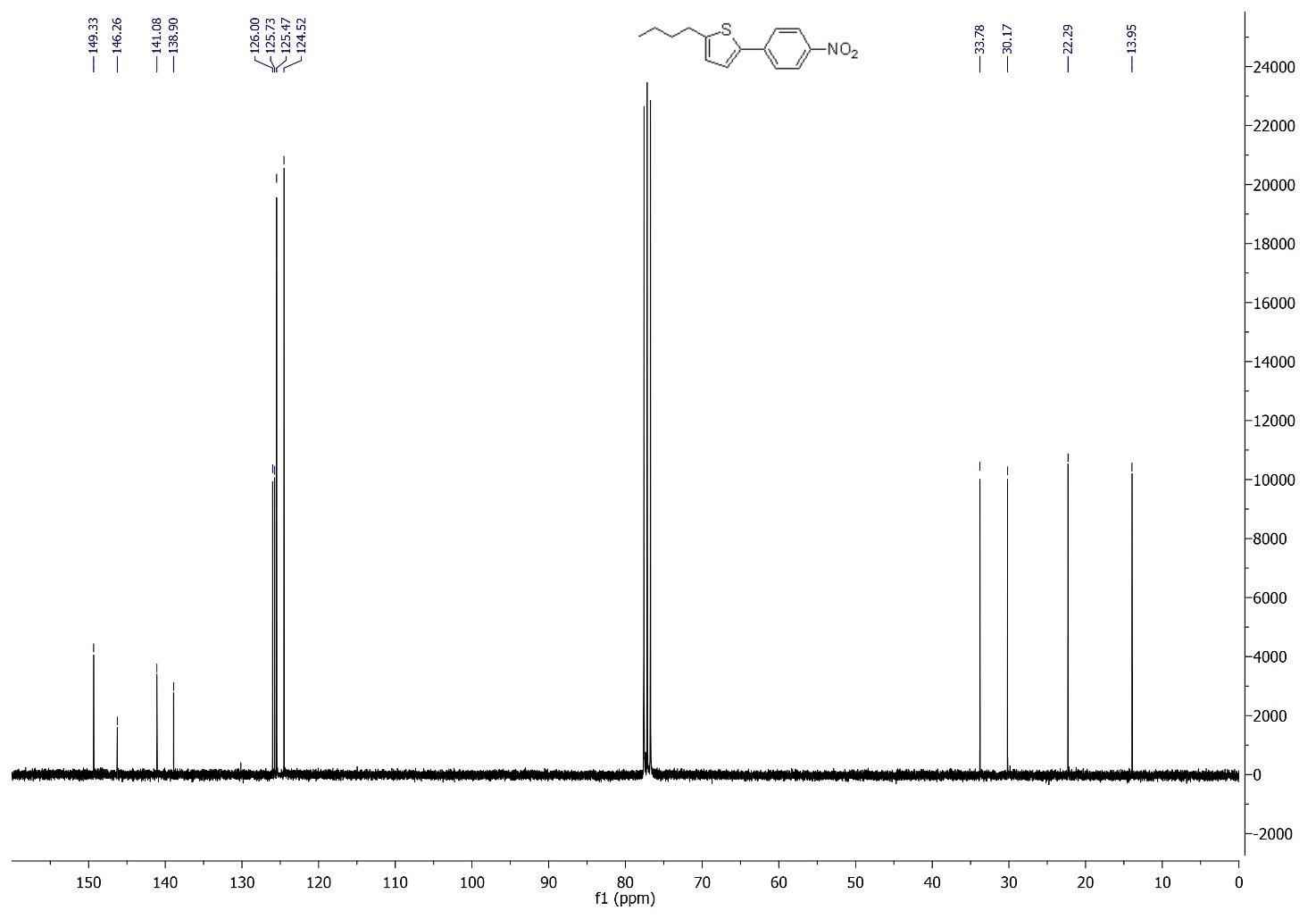
**

**1-(5-(4-nitrophenyl)thiophen-2-yl)ethenone (3c)**

**^1^H NMR (600 MHz, CDCl_3_)**: δ 8.28 (d, *J* = 9 Hz, 2H), 7.80 (d, *J* = 9 Hz, 2H), 7.69 (d, *J* = 3,9 Hz, 1H), 7.46 (d, *J* = 3,9 Hz, 1H), 2.59(s, 3H) ppm; **^13^C NMR (150 MHz, CDCl_3_)**: δ 190.6, 149.1, 147.7, 145.5, 139.4, 133.4, 126.8, 126.3, 124.6, 26.8 ppm.


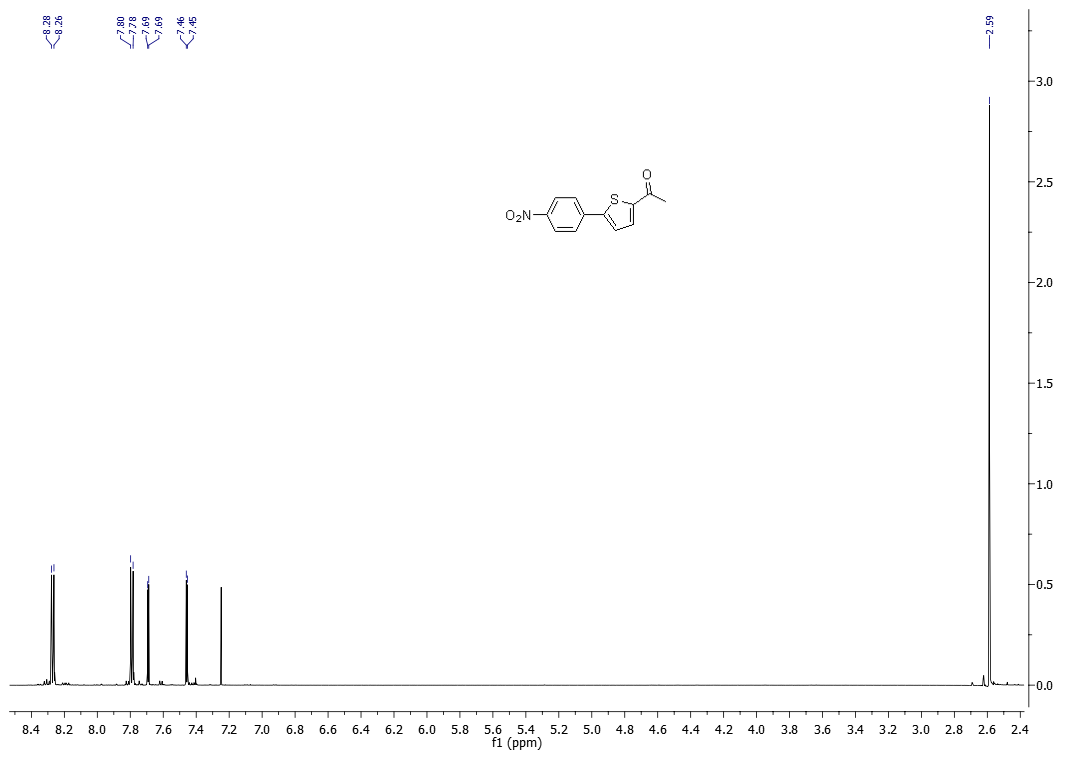


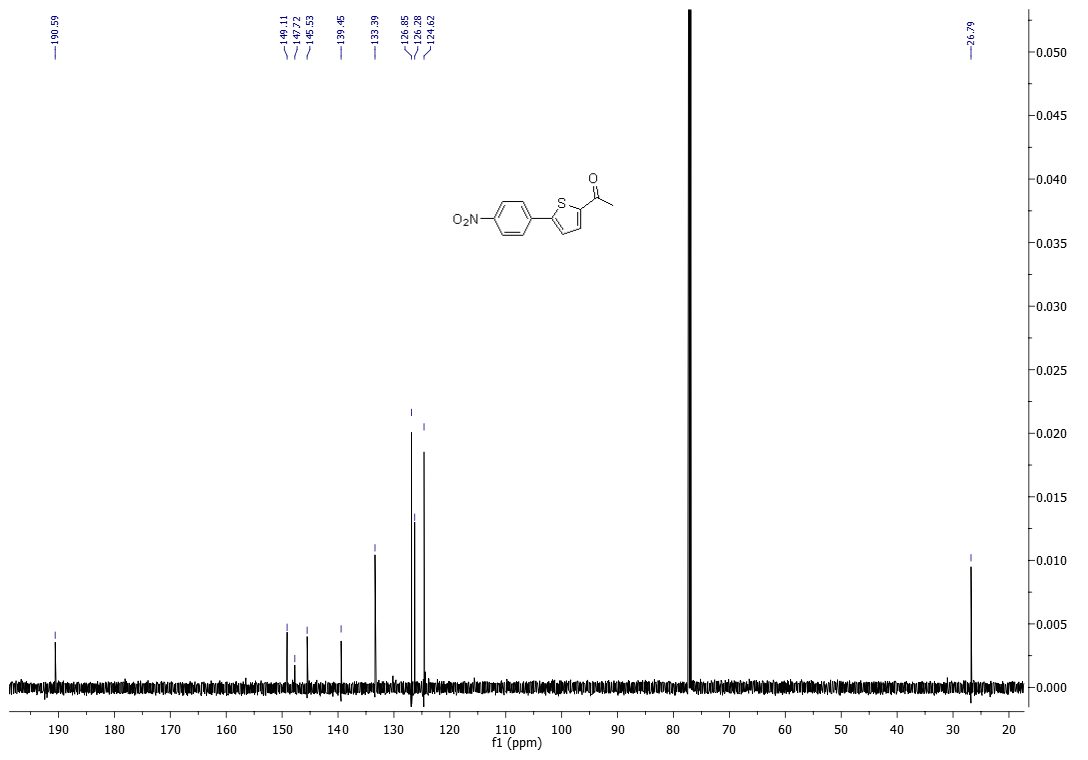


**2-methyl-5-(3-nitrophenyl)thiophene (3d)**

**^1^H NMR (600 MHz, CDCl_3_)**: δ 8.37 (t, J=2, 1H); 8.04 (dq, J=8.2 Hz, 0.9 Hz, 1H); 7.82 (dq, *J* = 8.2 Hz, 0.9 Hz, 1H), 7.5 (T, *J* = 7.9 Hz, 1H), 7.25 (d, J=3.5 1H), 6.77 (m, 1H), 2.52 (d, J=1, 3H) ppm; **^13^C NMR (150 MHz, CDCl_3_)**: δ 148.8, 141.6, 139.1, 136.4, 131.1, 129.8, 126.8, 124.8, 121.4, 120.0, 15.6 ppm.


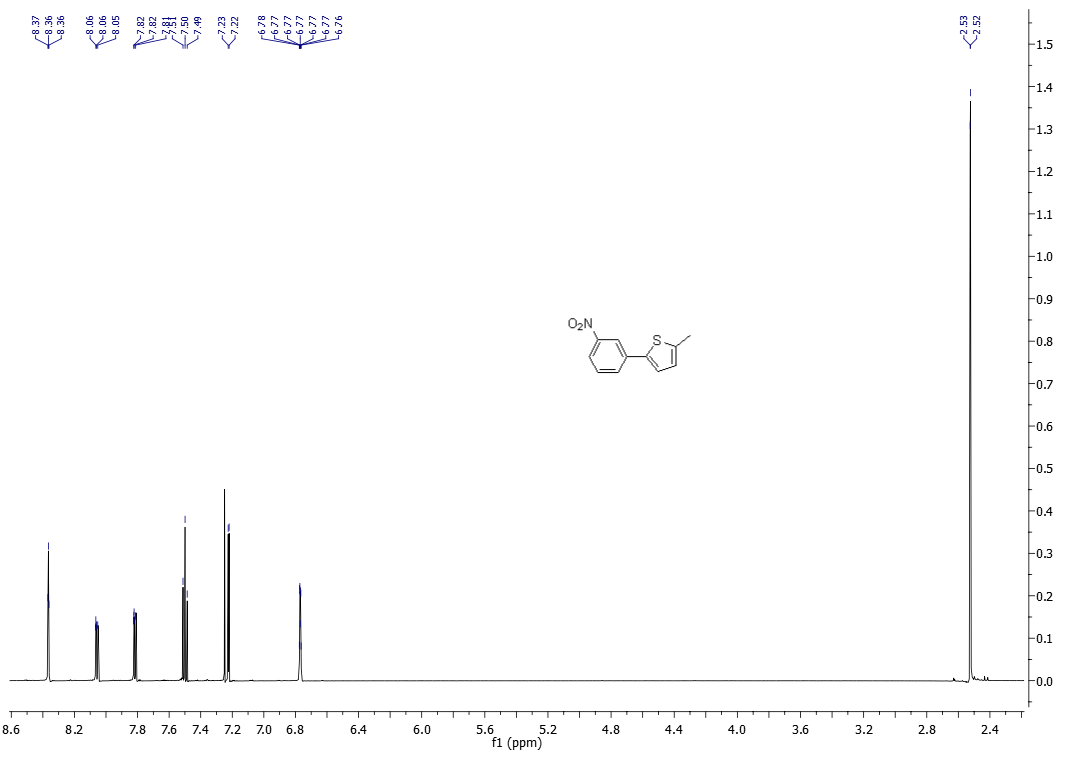


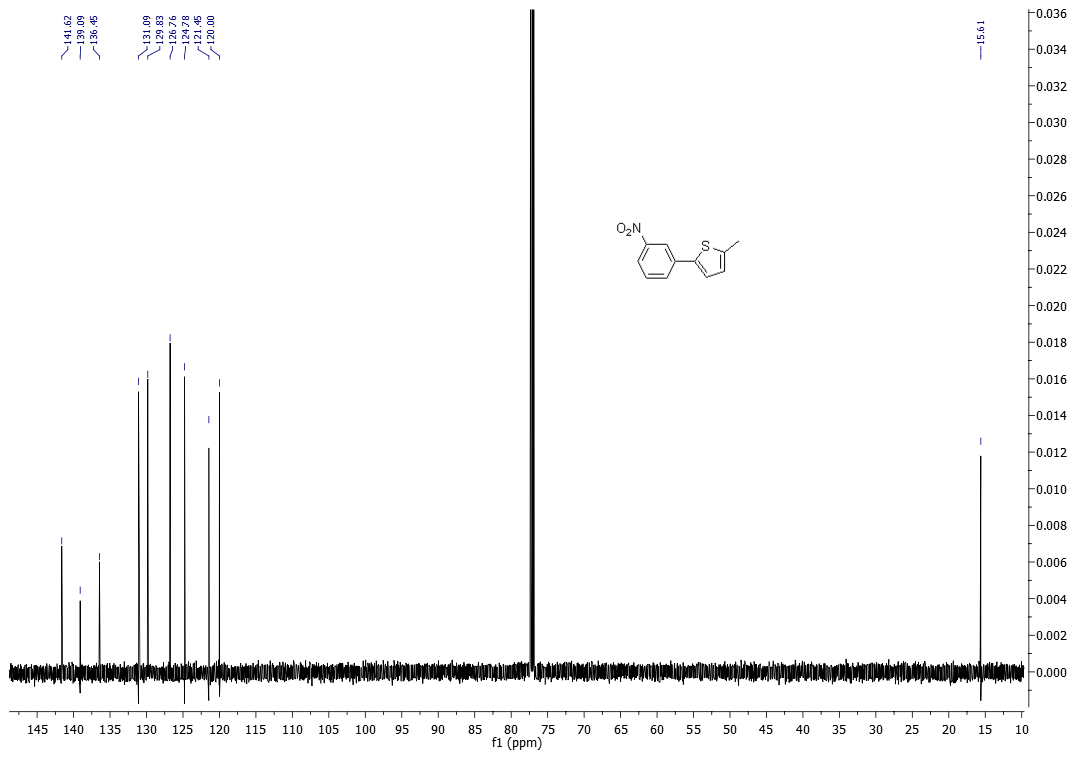


**1-(4-(5-methylthiophen-2-yl)phenyl)ethenone (3e)**

**^1^H NMR (600 MHz, CDCl_3_)**: δ 7.87 (d, *J* = 9 Hz, 2H), 7.54 (d, *J* = 9 Hz, 2H), 7.16 (d, *J* = 3 Hz, 1H), 6.70 (m, 1H), 2.53 (s, 3H), 2.45 (s, 3H) ppm; **^13^C NMR (150 MHz, CDCl_3_)**: δ 197.5, 141.7, 140.6, 139.2, 135.4, 129.2, 126.8, 125.2, 124.7, 26.7, 15.7 ppm.


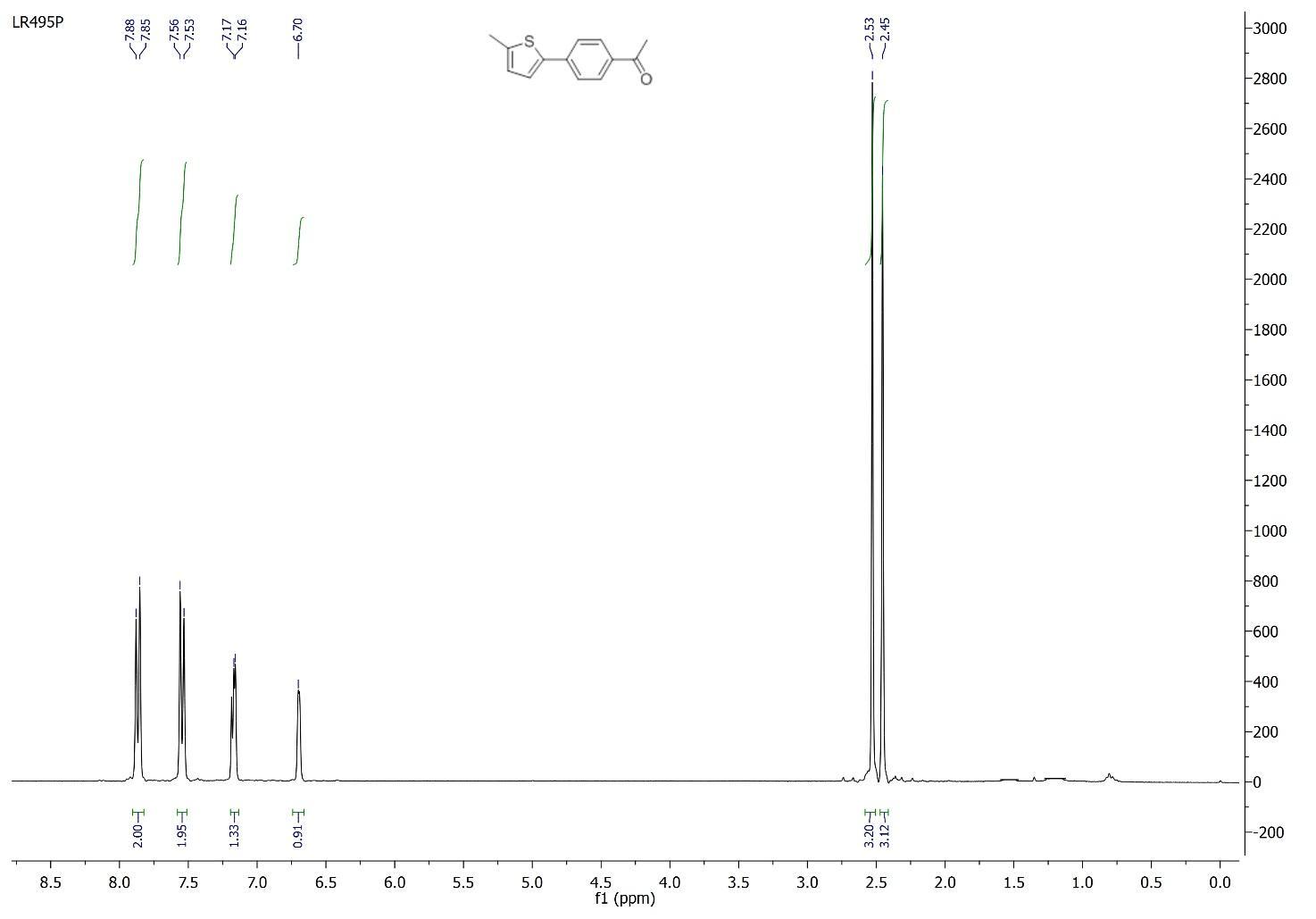


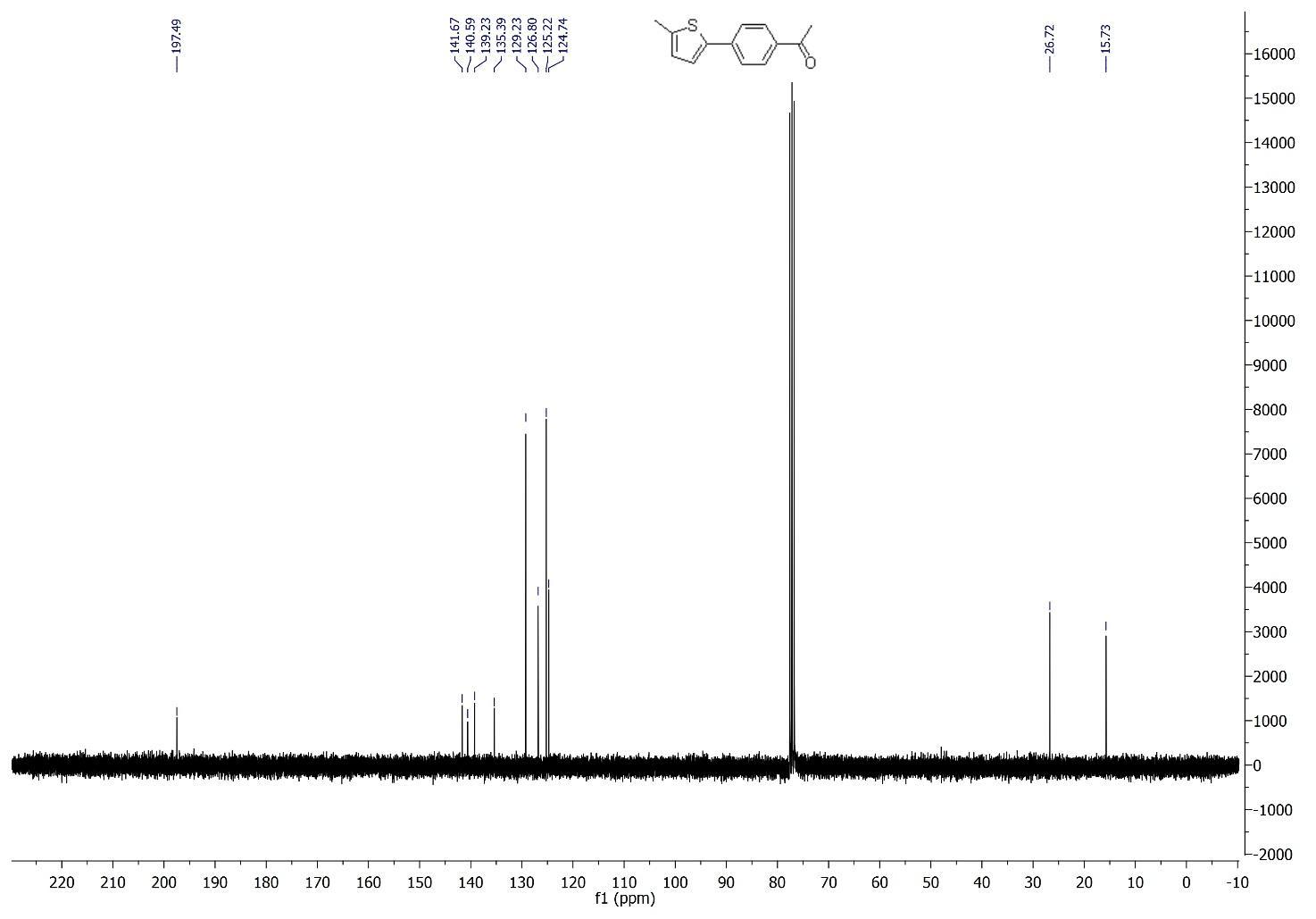


**1-(3-(5-methylthiophen-2-yl)phenyl)ethenone (3f)**

**^1^H NMR (600 MHz, CDCl_3_)**: δ 8.12 (m, 1H), 7.81 (d, *J* = 9 Hz, 1H), 7.73 (d, *J* = 6 Hz, 1H), 7.45 (dd, *J* = 9 Hz, 6 Hz, 1H), 7.18 (d, *J* = 3 Hz, 1H), 6.75 (d, *J* = 3 Hz, 1H), 2.64 (s, 3H), 2.52 (s, 3H) ppm; **^13^C NMR (150 MHz, CDCl_3_)**: δ 197.98, 140.69, 140.37, 137.61, 135.20, 129.88, 129.08, 126.78, 126.39, 125.02, 123.70, 26.75, 15.50 ppm.


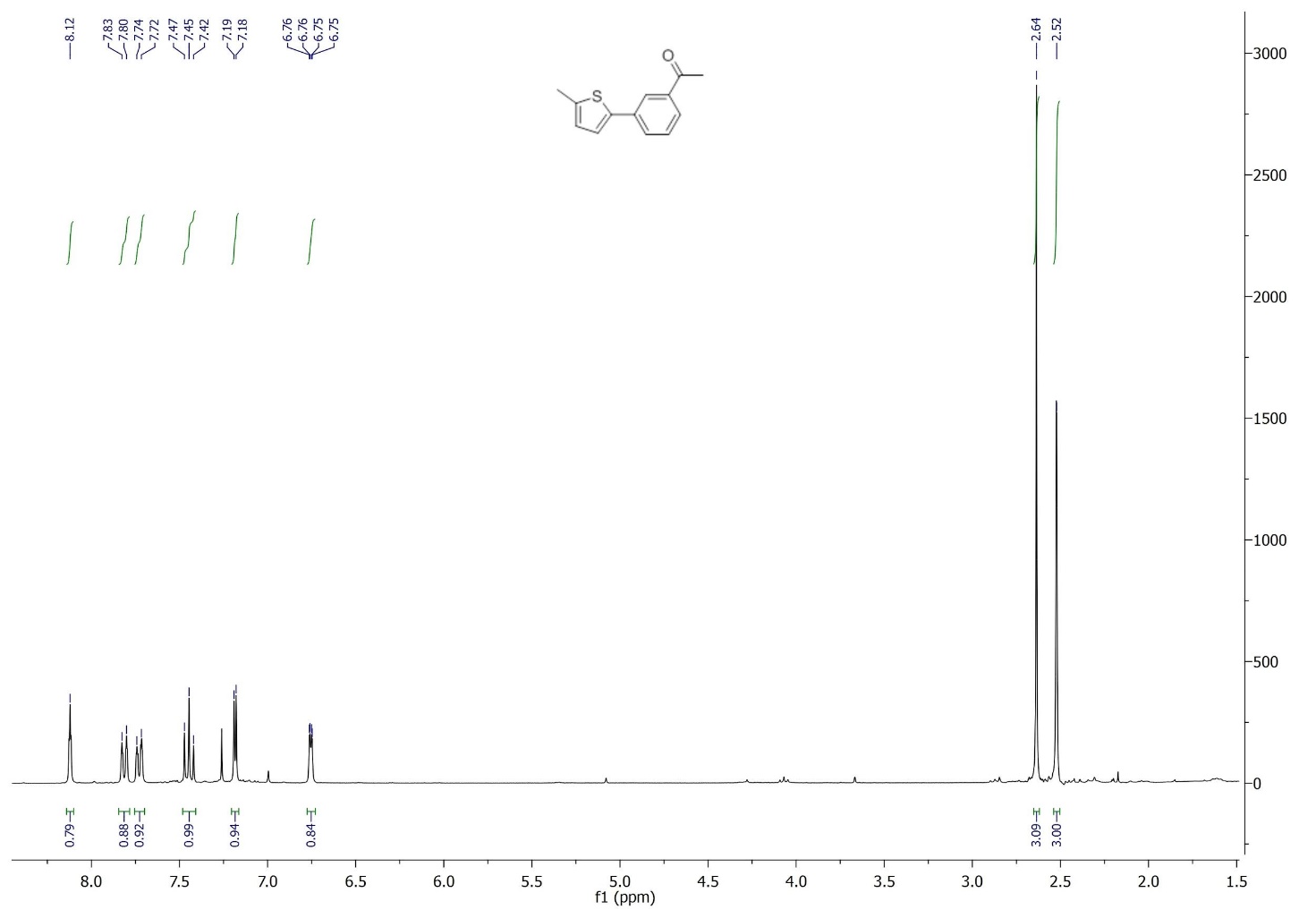


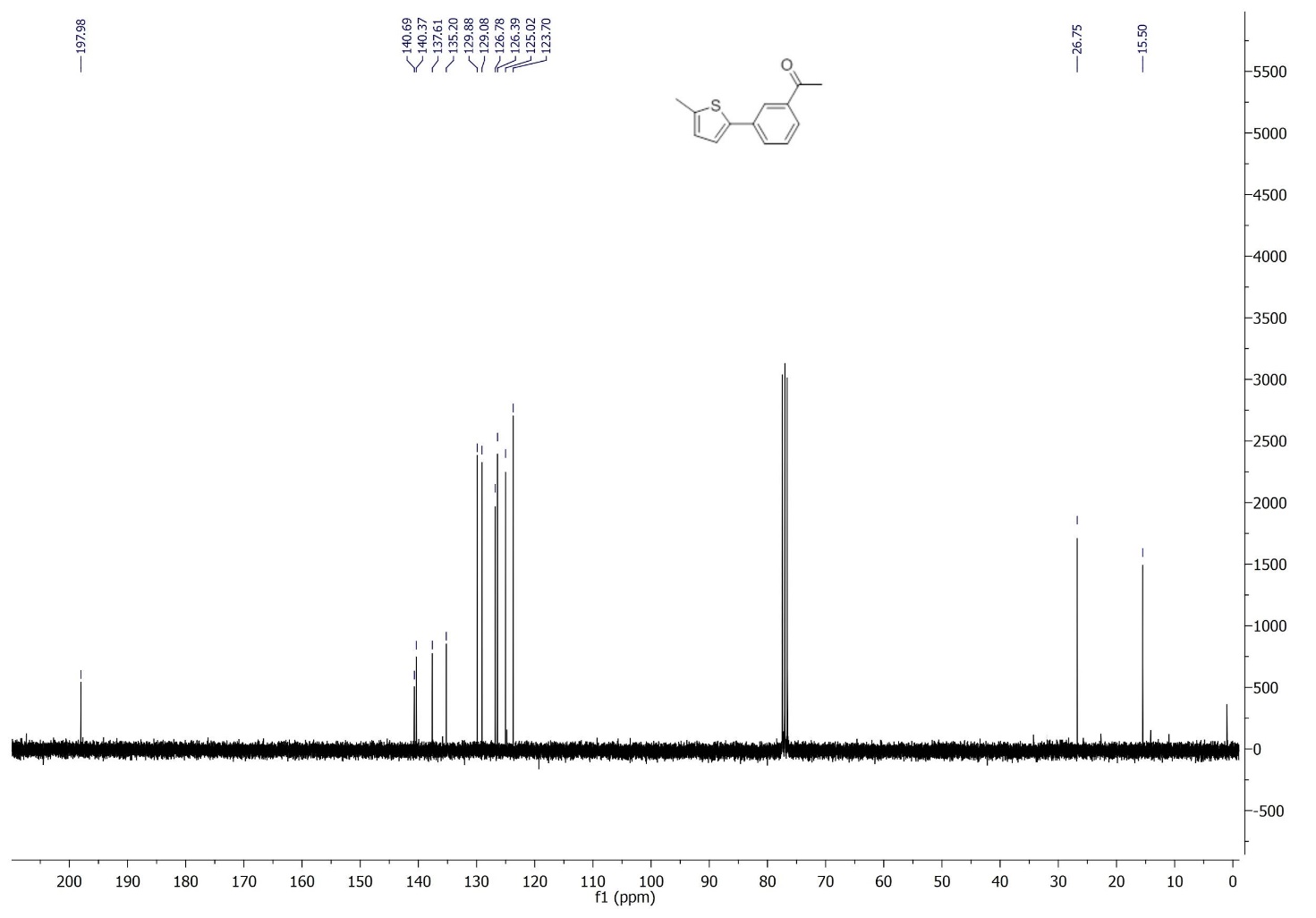


**1-(4-(5-butylthiophen-2-yl)phenyl)ethenone (3h)**

**^1^H NMR (600 MHz, CDCl_3_)**: δ 7.95 (d, *J* = 9 Hz, 2H), 7.63 (d, *J* = 9 Hz, 2H), 7.25 (d, J = 3 Hz, 1 H) 6.78 (d, *J* = 3 Hz, 1H), 2.84 (t, J = 9 Hz, 2H), 2.60 (s, 3H), 1.75-1.64 (m, 2H), 1.43-1.40 (m, 2H), 0.95 (t, J = 6 Hz, 3H) ppm; **^13^C NMR (150 MHz, CDCl_3_)**: δ 197.2, 147.5, 139.8, 136.0, 135.1, 125.5, 124.9, 124.4, 33.6, 26.4, 22.0, 13.7 ppm.


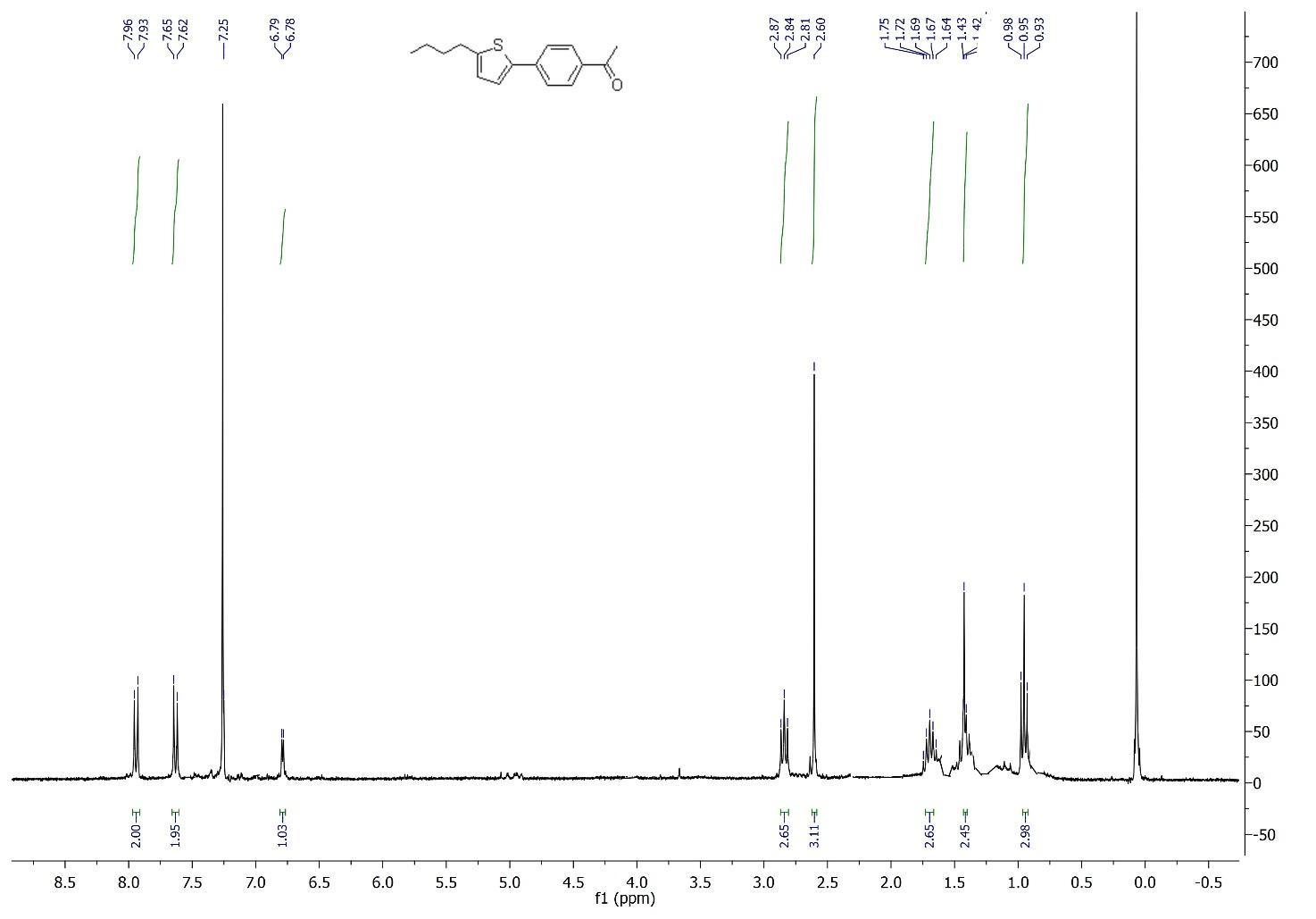


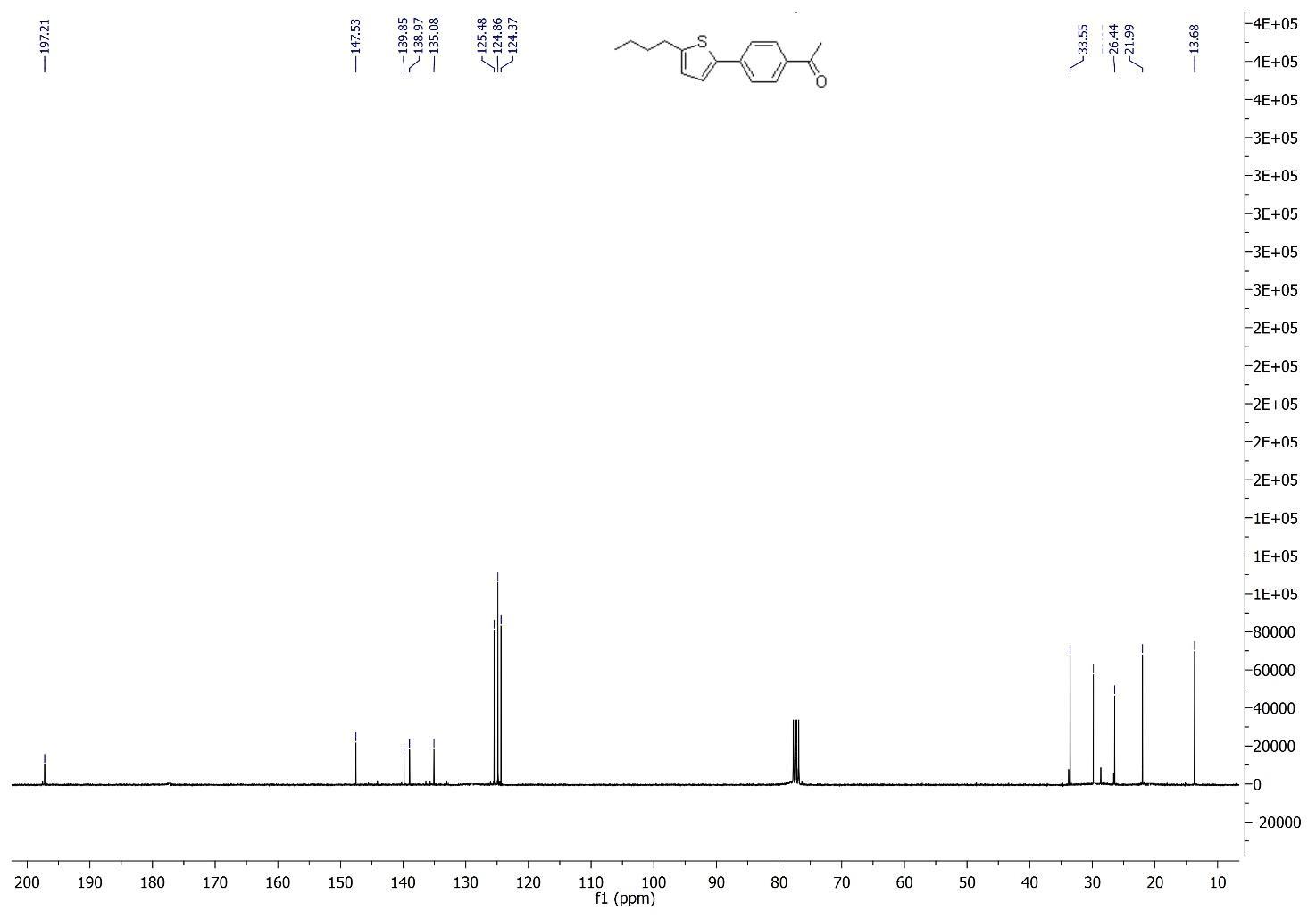


**4-(5-methylthiophen-2-yl)benzonitrile (3l)**

**^1^H NMR (**600 MHz, CDCl_3_**)**: δ 7.60 (s, 4H); 7.20 (d, J=4, 1H); 6.76 (dd, J=4 Hz, 1 Hz, 1H); 2.51 (d, J=1 3H) ppm. **^13^C NMR** (150 MHz, CDCl_3_): δ 142.9, 141.1, 139.3, 131.4, 127.1, 126.0, 125.3, 119.2, 110.1.5, 15.7 ppm.


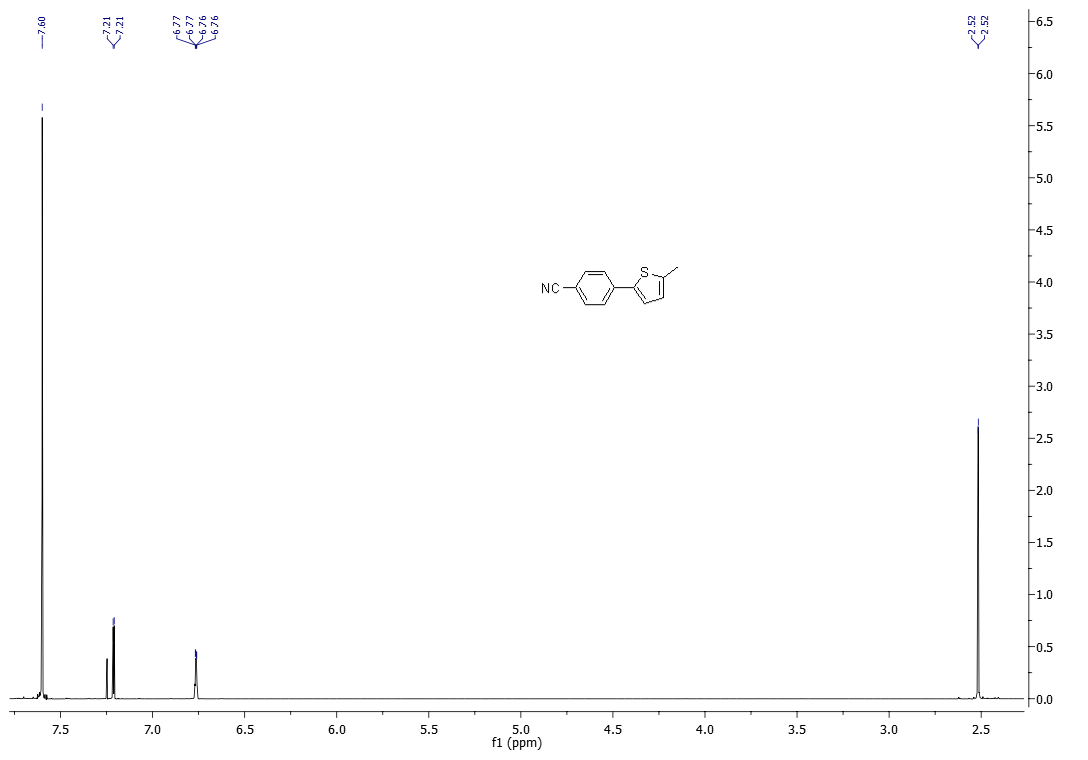


**
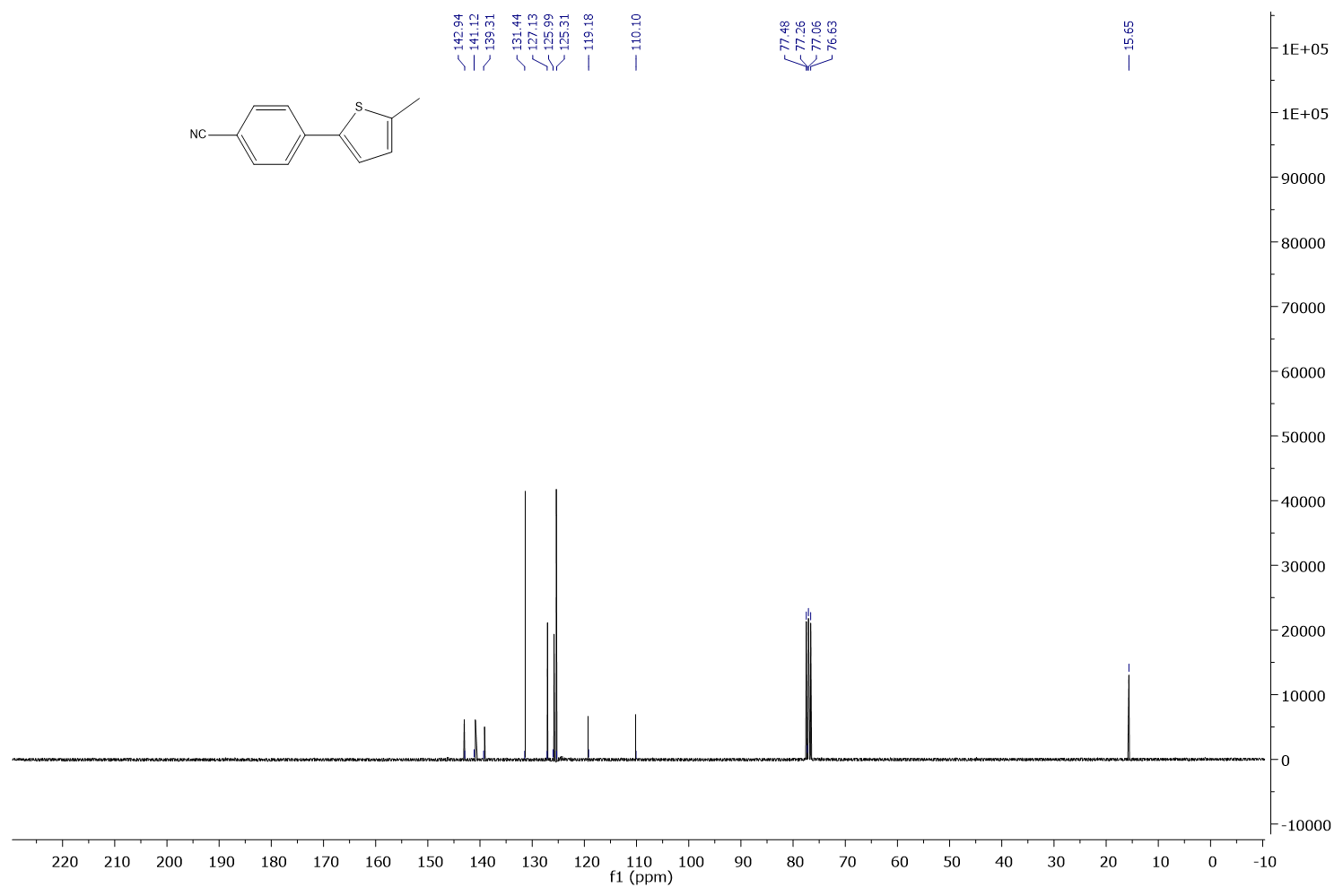
**

**4-(5-acetylthiophen-2-yl)benzonitrile (3m)**

**^1^H NMR (600 MHz, CDCl_3_)**: δ 7.78 (d, *J* = 8.5 Hz, 2H), 7.61 (d, J = 3.9 Hz, 1H), 7.61 (d, *J* = 8,5 Hz, 2H), 7.45 (d, *J* = 3,9 Hz, 1H), 2.58(s, 3H) ppm; **^13^C NMR (150 MHz, CDCl_3_)**: δ 190.4, 149.6, 145.0, 137.5, 133.2, 133.9, 126.6, 125.7, 118.4, 112.2, 26.6 ppm.

**
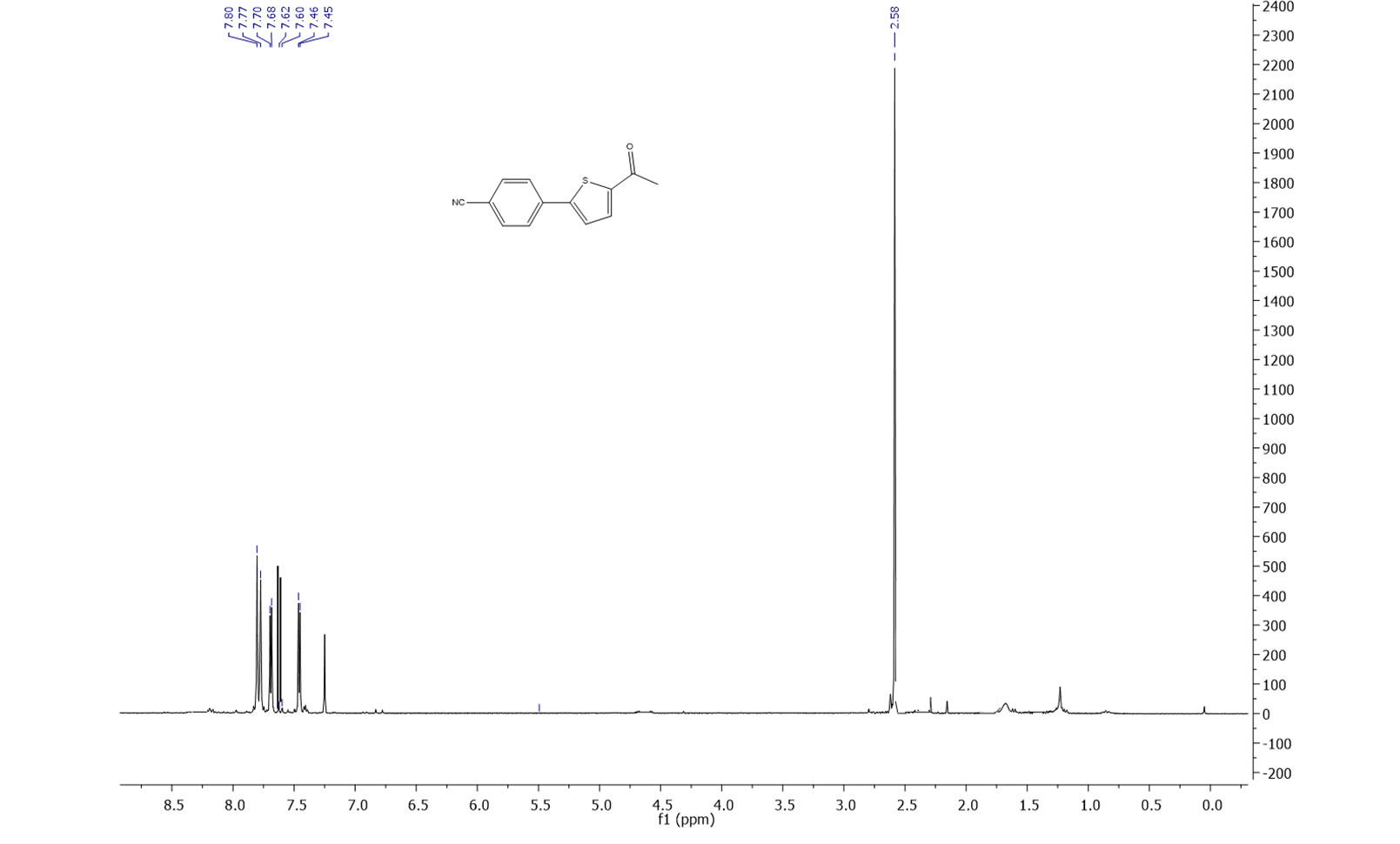
**

**
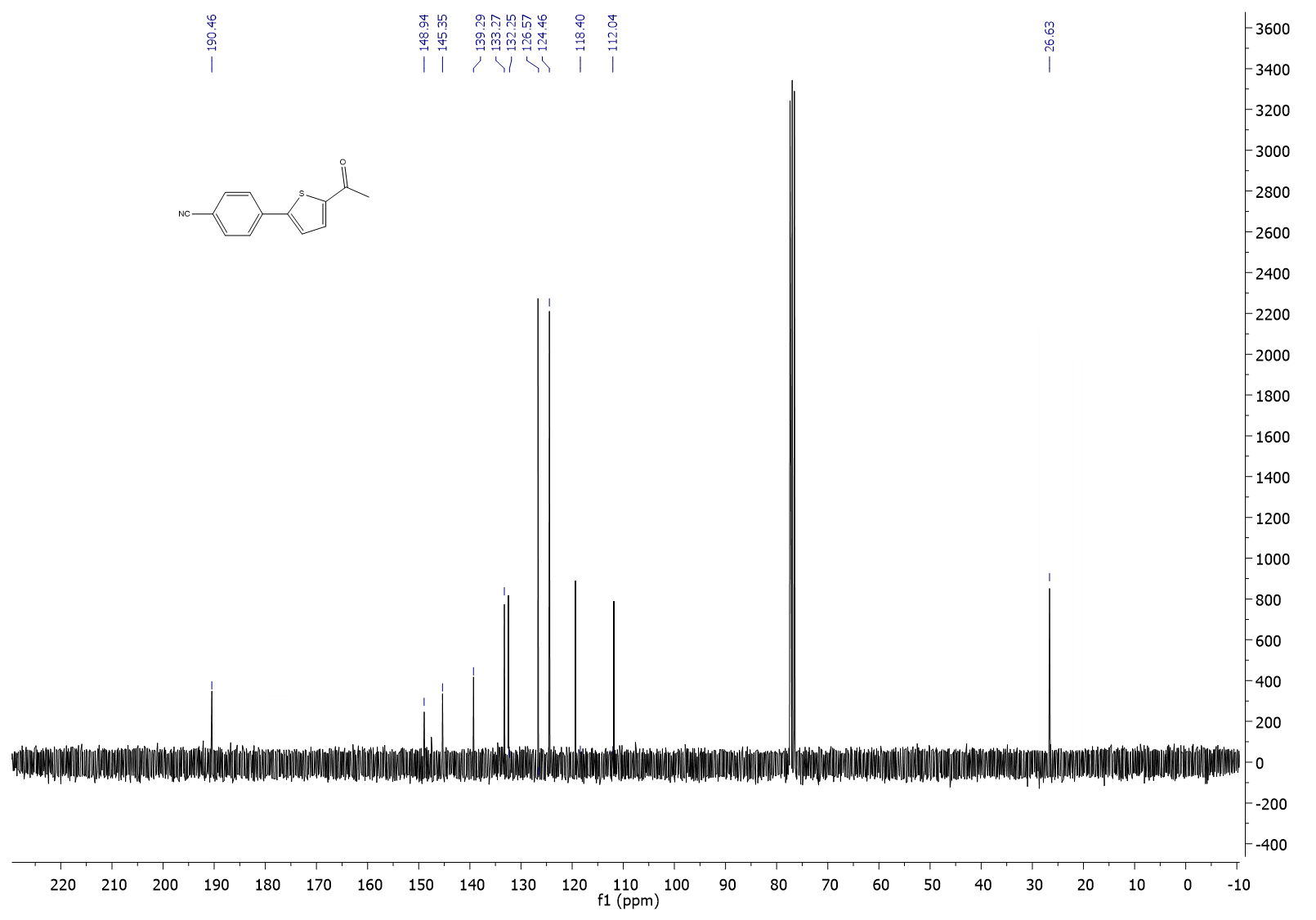
**

**4-(5-butylthiophen-2-yl)benzonitrile (3n)**

**^1^H NMR (600 MHz, CDCl_3_)**: δ 7.58 (s, 4H); 7.21 (d, J=3.6, 2H); 6.77 (d, J=3.6 Hz, 1H), 2.82 (t, J=7.5 Hz, 2H); 1.69 (q, J=1.5, 2H); 1.42 (q, J=1.5, 2H); (t, J=7.5, 1H) **^13^C NMR (150 MHz, CDCl_3_)**: δ 148.5, 139.2, 133.5, 133.4, 132.7, 125.7, 125.5, 125, 119.1, 109.9, 33.7, 30.1, 22.2, 13.9 ppm.


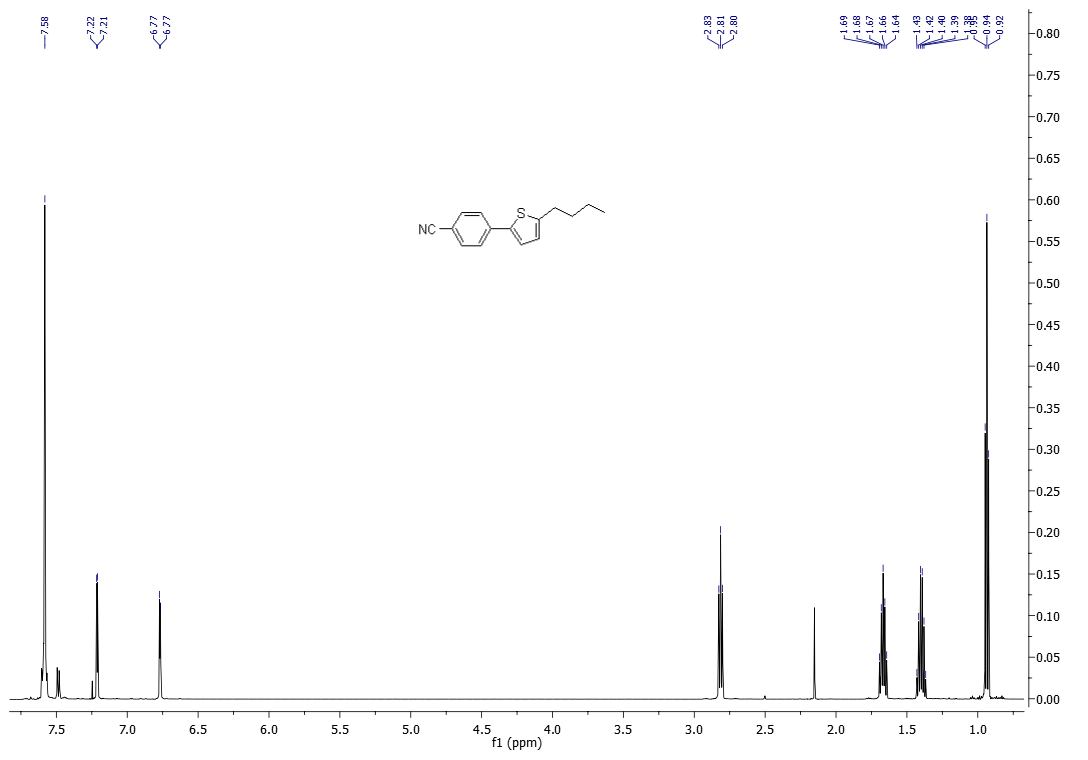


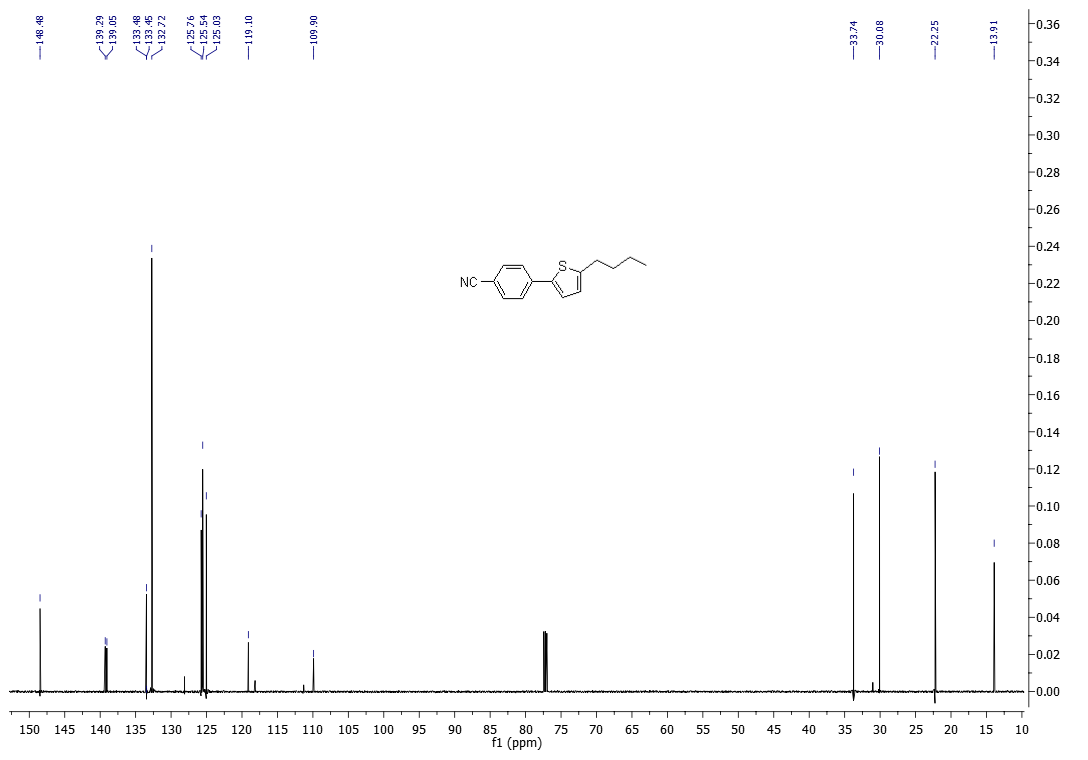


**2-methyl-5-(4-(trifluoromethyl)phenyl)thiophene (3o)**

**^1^H NMR (600 MHz, CDCl_3_)**: δ 7.63 (d, J=8.3, 2H); 7.61 (d, J=8.3, 2H); 7.18 (d, J=3.6 Hz, 1H), 6.76 (dd, J=3.6 Hz, 1 Hz, 1H), 2.52 (d, J=1, 3H) ppm; **^13^C NMR (150 MHz, CDCl_3_)**: δ 141.3, 140.2, 138.1, 126.6, 125.93, 125.9, 125.88, 125.85, 125.5, 124.4, 123.4, 15.6 ppm.


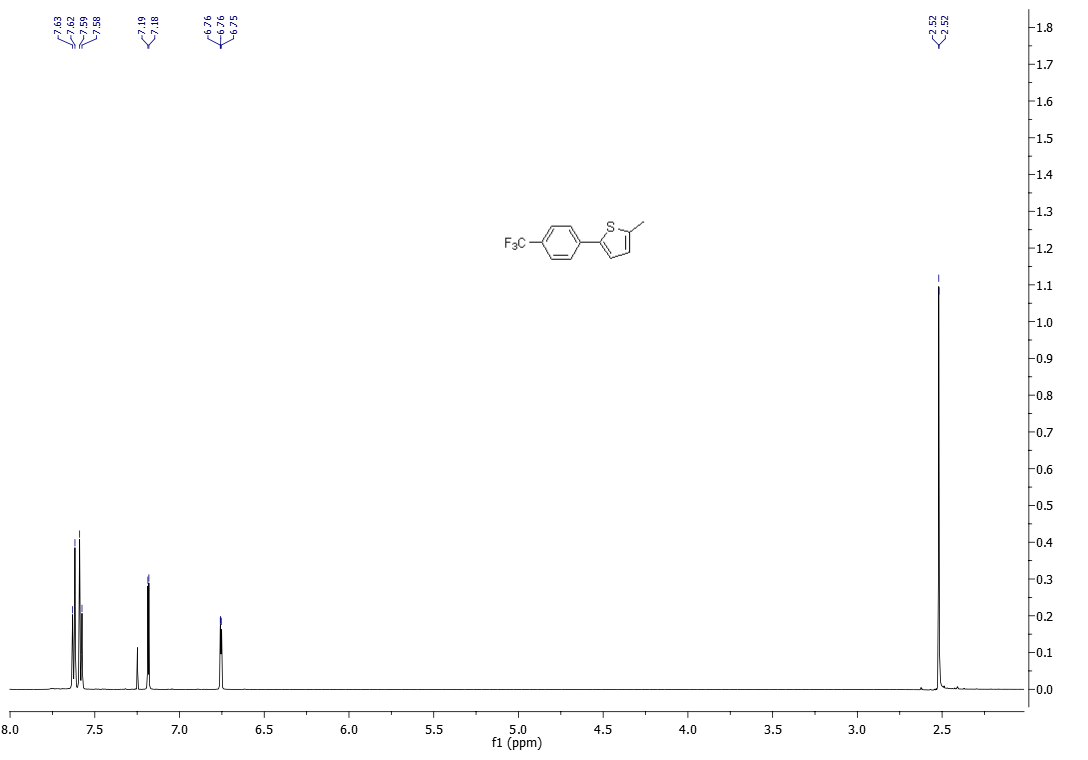


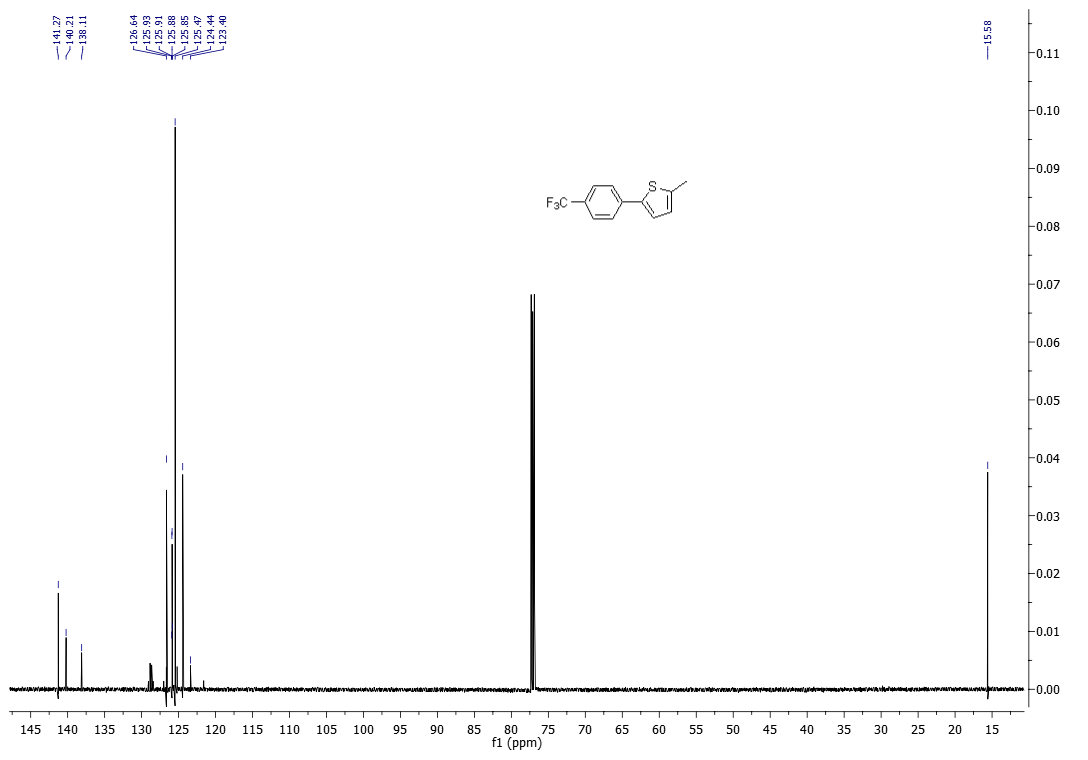


**1-(5-(4-(trifluoromethyl)phenyl)thiophen-2-yl)ethenone (3p)**

**^1^H NMR** (600 MHz, CDCl_3_)= 7.73 (2H, d, J=8.2); 7.66 (1H, s); 7.65 (2H, d, J=8.2); 7.38 (1H, d, J=3.9); 2.57 (3H, s).**^13^C-NMR:** (150 MHz, CDCl_3_): 190.6; 150.4; 144.4; 136.8; 136.7; 133.4; 126.5; 126.22; 126.20; 125.23; 124.8; 123.1; 26.7.


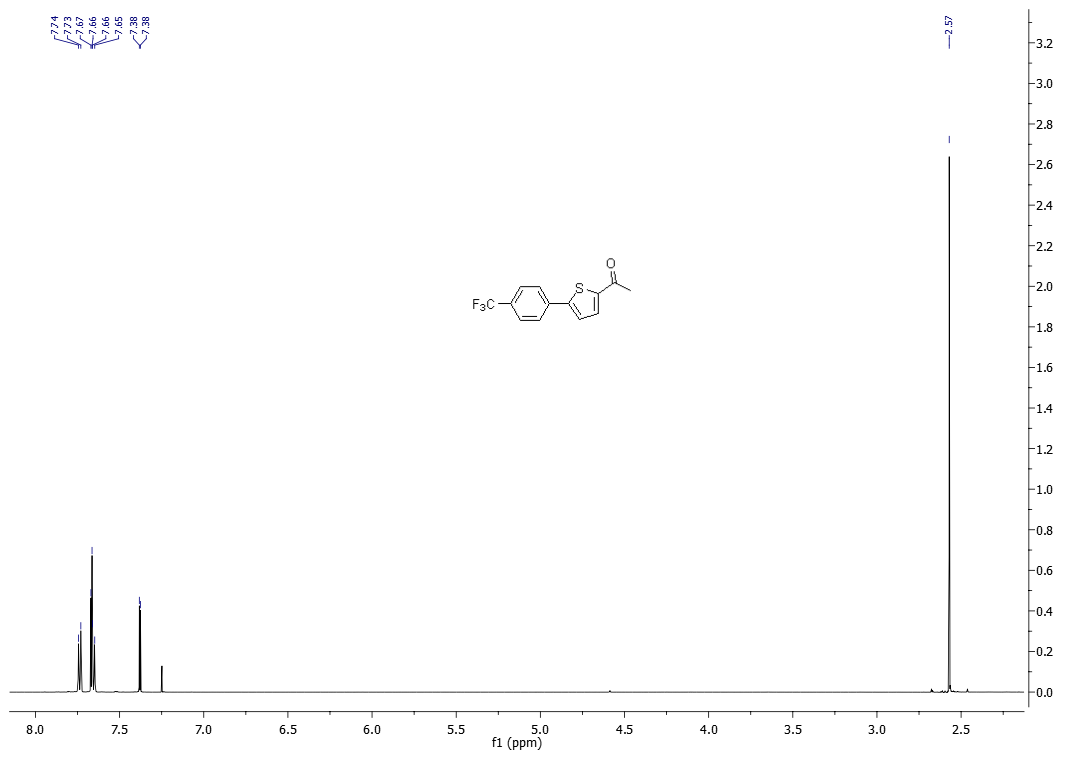


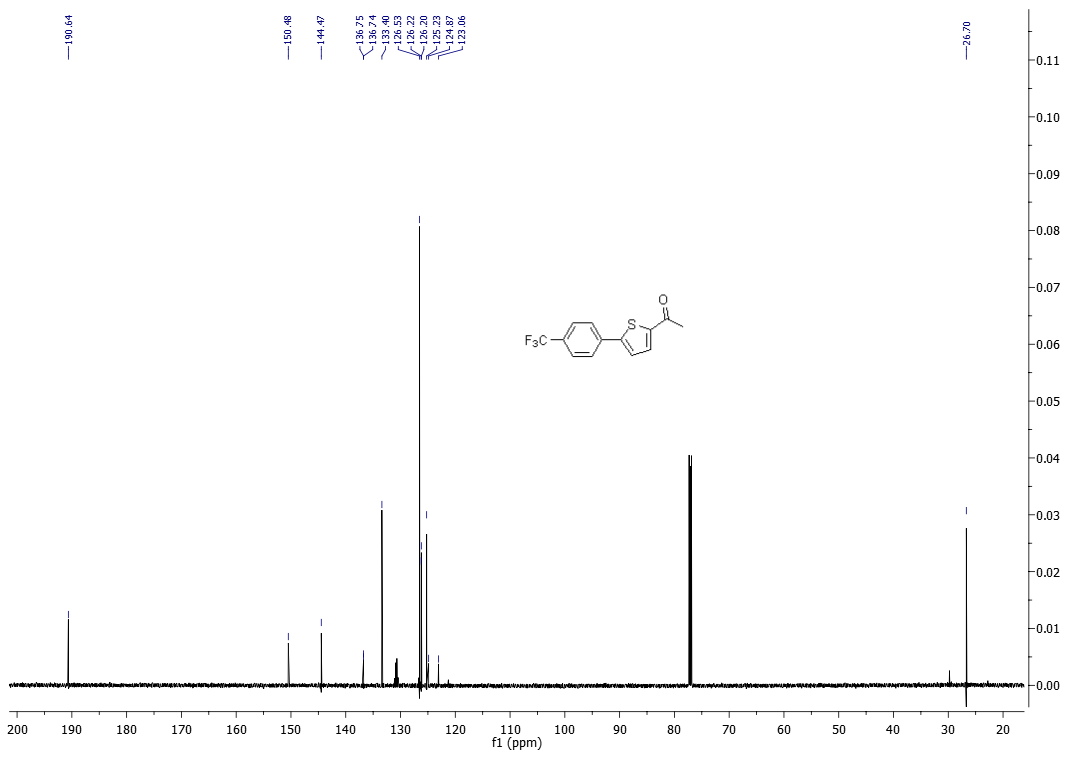


**2-butyl-5-(4-(trifluoromethyl)phenyl)thiophene (3q)**

**^1^H NMR (600 MHz, CDCl_3_)**: δ 7.84 (d, J = 8.4 Hz, 2H); 7.58 (d, J=8.4Hz, 2H); 7.22 (d, J=3.6 Hz, 1H), 6.73 (d, J = 3.6 Hz, 1H), 2.77 (t, J=7.5 Hz, 2H); 1.62 (q, J=1.5, 2H); 1.36 (q, J=1.5, 2H); 0.88 (t, J=7.5, 1H) **^13^C NMR (150 MHz, CDCl_3_)**: δ 147.7, 144.2, 139.3, 137.2, 131.2, 129.6, 124.1, 123.9, 123.0, 31.8, 28.6, 20.9, 11.9 ppm.

**
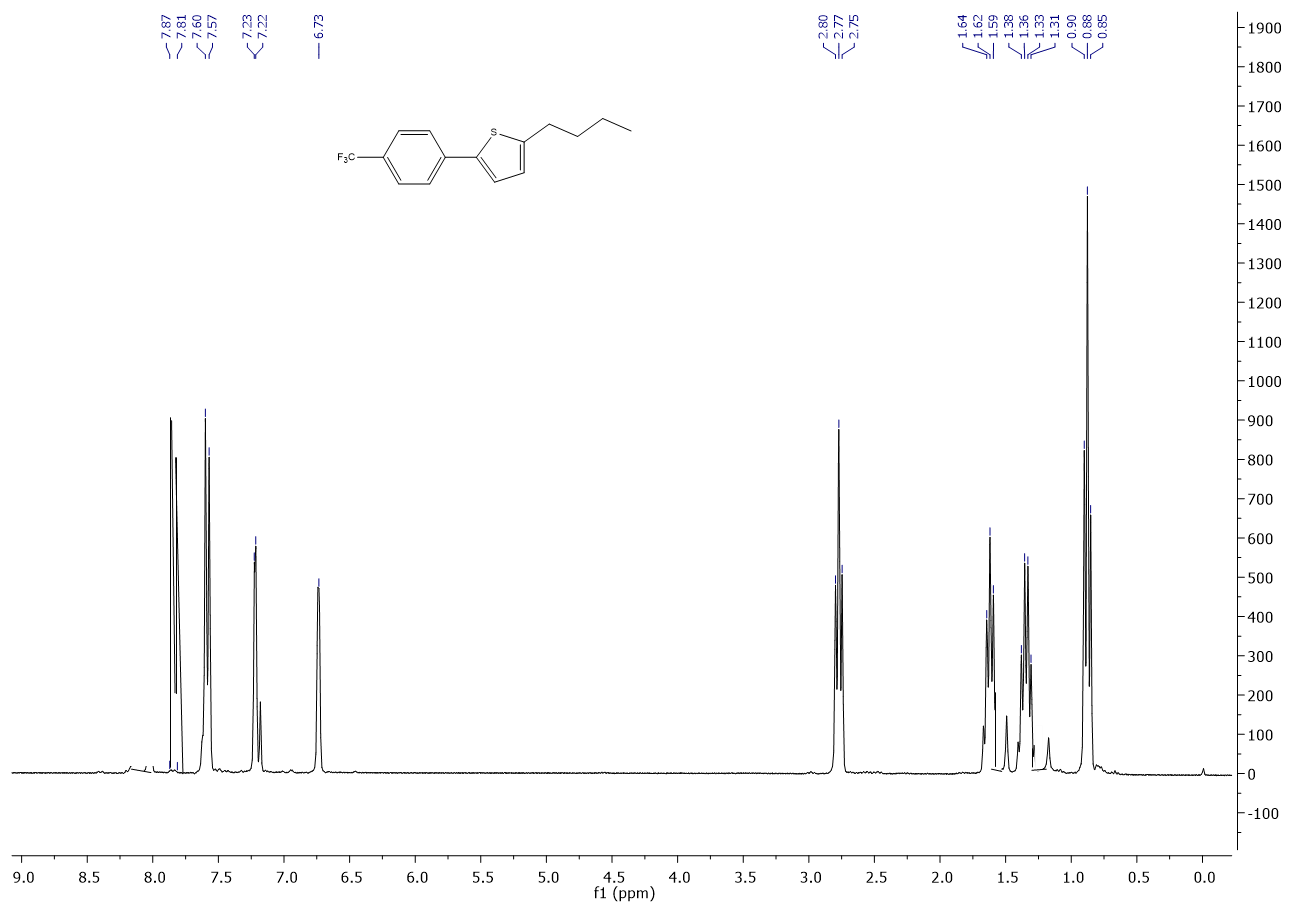
**

**2-(4-methoxyphenyl)-5-methylthiophene (3r)**

**^1^H NMR (600 MHz, CDCl_3_)**: δ 7.63 (d, *J* = 9 Hz, 2H), 6.98 (d, *J* = 3 Hz, 1H), 6.89 (d, *J* = 9 Hz, 2H), 6.70 (m, 1H), 3.83 (s, 3H), 2.49 (s, 3H), ppm; **^13^C NMR (150 MHz, CDCl_3_)**: δ 159.2, 142.3, 138.9, 128.0, 127.1, 126.4, 122.2, 114.6, 55.8, 15.8 ppm.


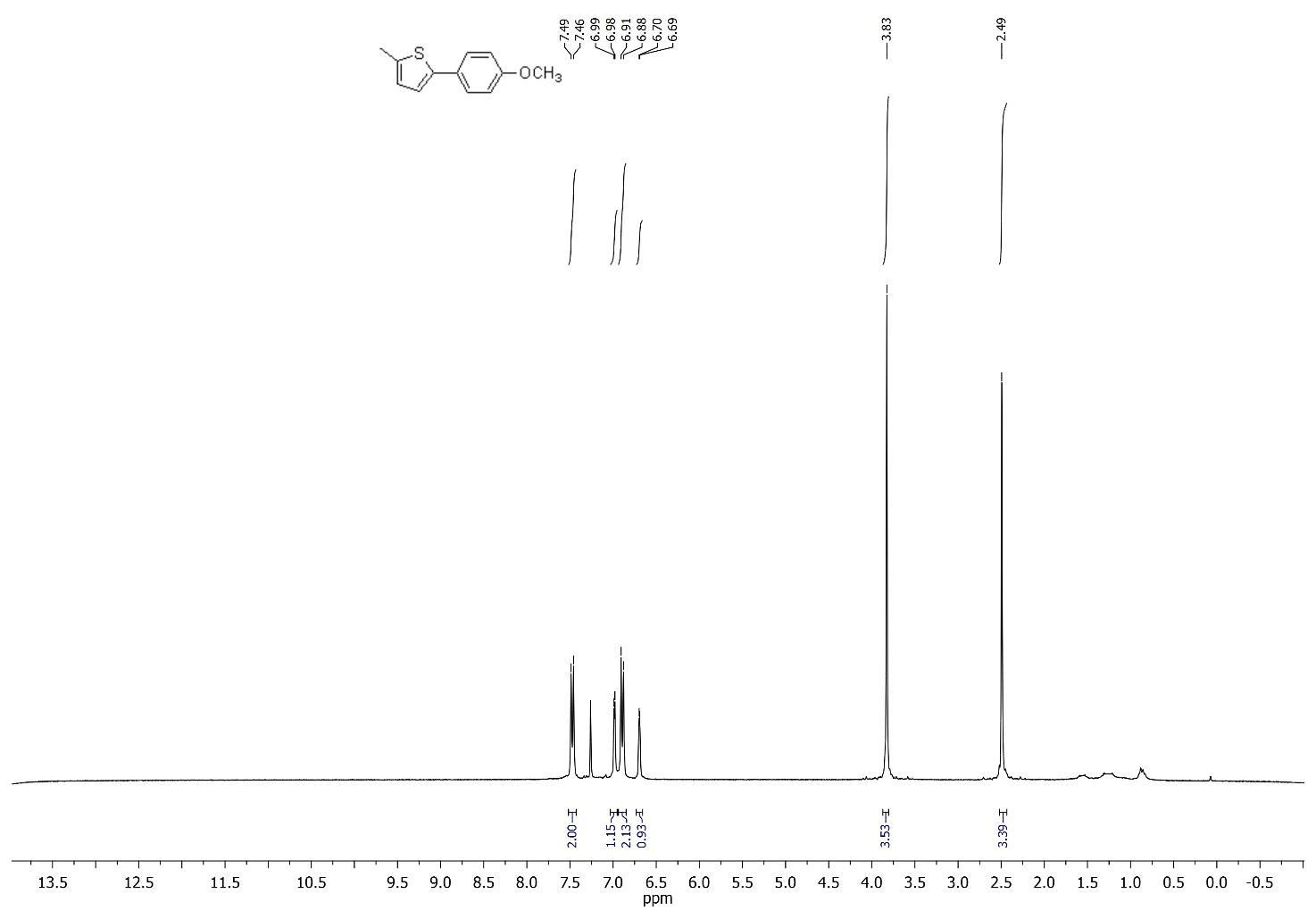


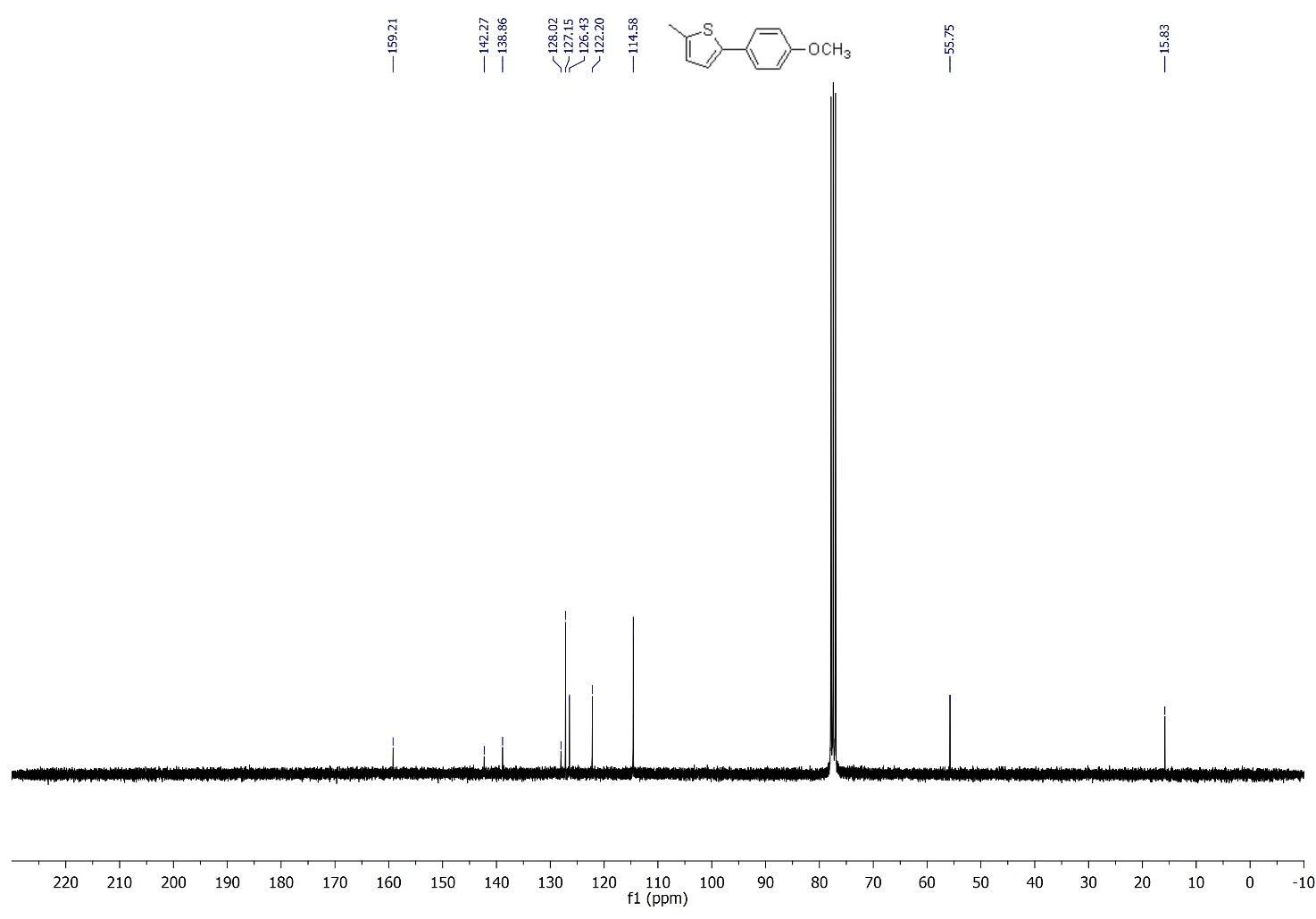


**2-butyl-5-(4-methoxyphenyl)thiophene (3u)**

**^1^H NMR (600 MHz, CDCl_3_)**: δ 7.48 (d, *J* = 9 Hz, 2H), 6.99 (d, *J* = 3 Hz, 1H), 6.88 (d, *J* = 9 Hz, 2H), 6.70 (d, 3 Hz, 1H), 3.82 (s, 3H), 2.80 (t, J = 7 Hz, 2H), 1.68 (m, 2H), 1.42 (m, 2H), 0.94 (t, J = 7Hz, 3H) ppm; **^13^C NMR (150 MHz, CDCl_3_)**: δ 158.9, 144.8, 141.7, 127.5, 126.9, 125.0, 121.7, 114.3, 55.5, 33.9, 30.0, 22.3, 14.0 ppm.


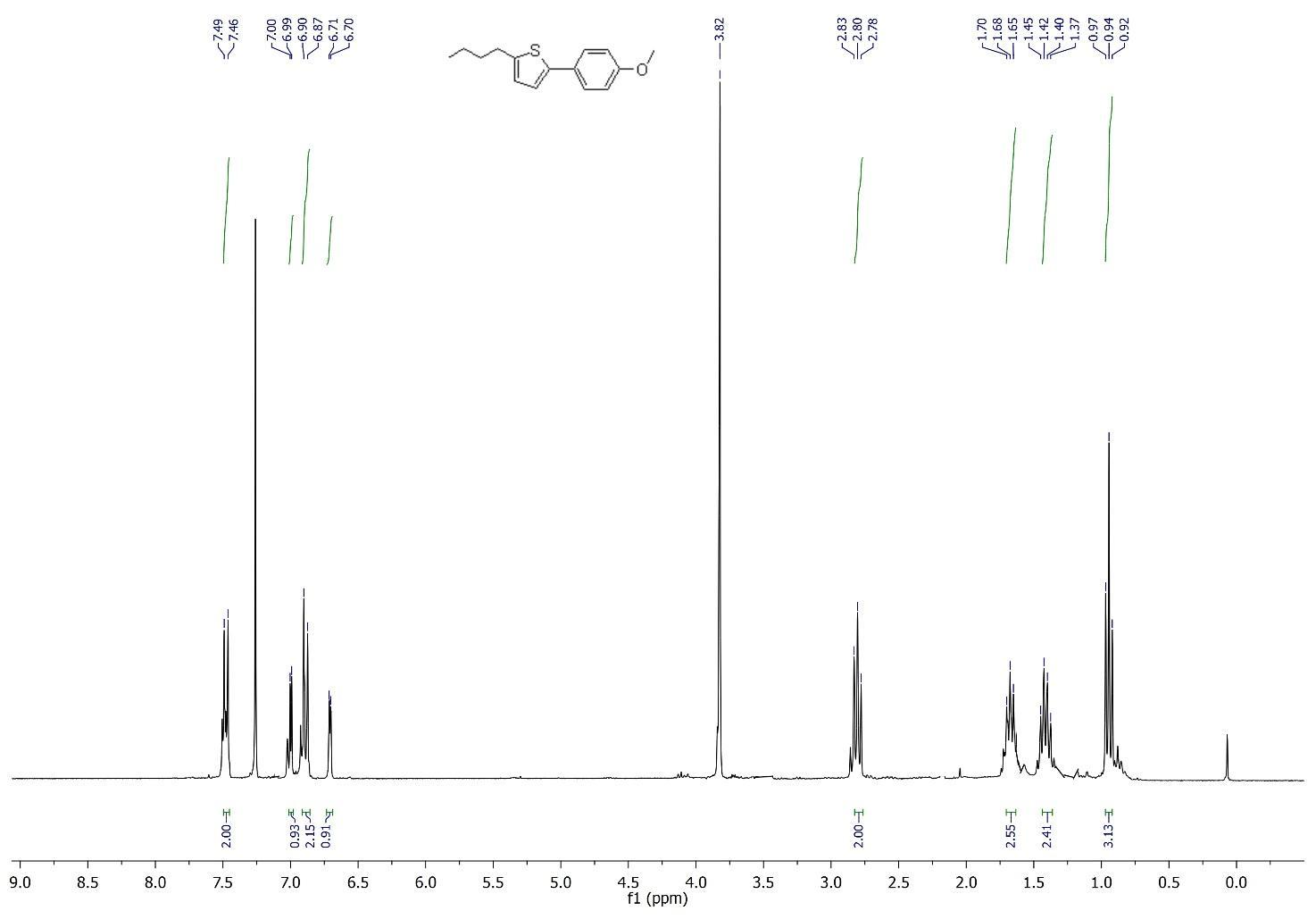


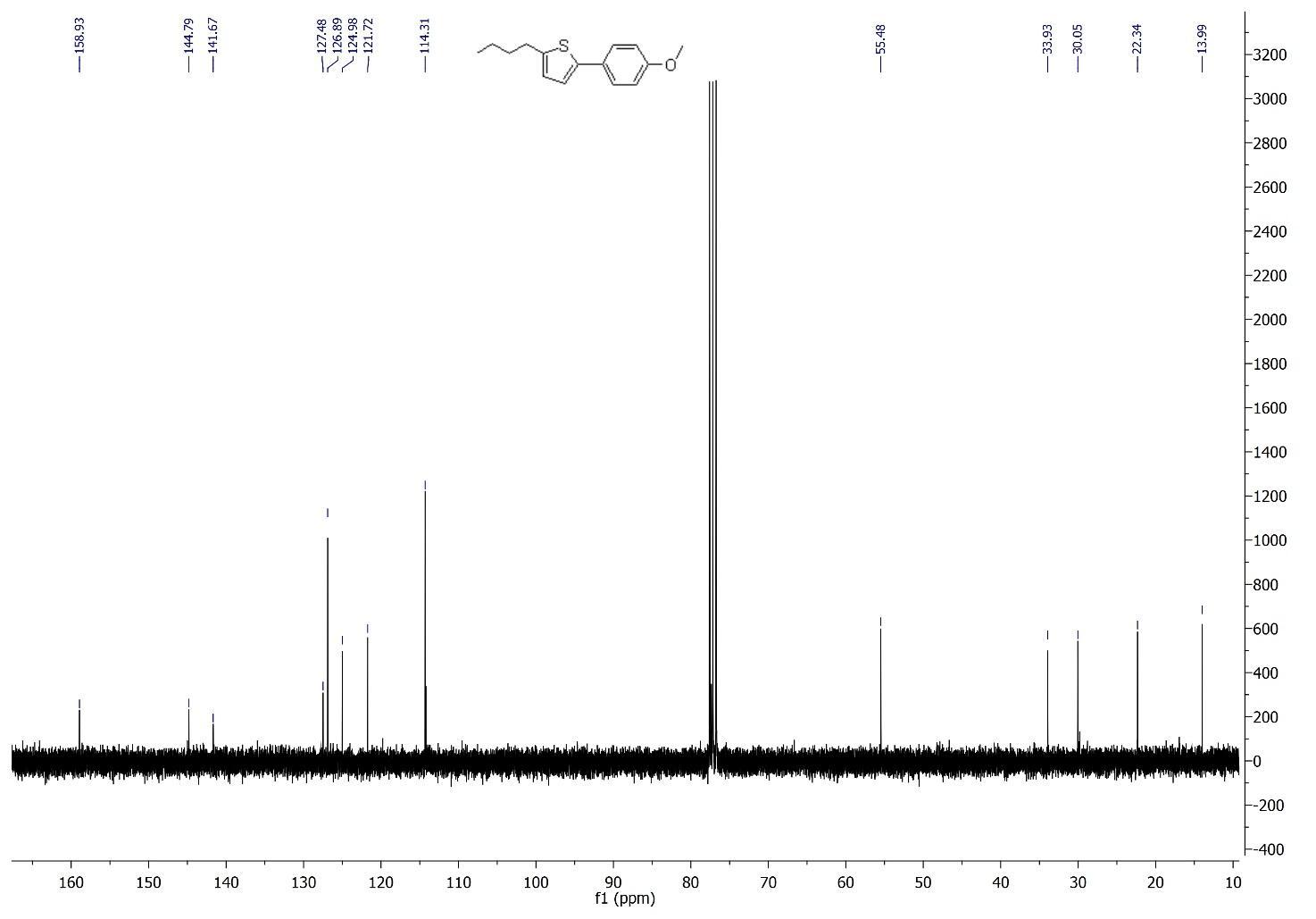


**2-methyl-5-(o-tolyl)thiophene (3v)**

**^1^H NMR (600 MHz, CDCl_3_)**: δ 7.39-7.36 (m, 1H), 7.24-7.19 (m, 3H), 6.85 (d, *J* = 3 Hz, 1H), 6.74 (m, 1H), 2.52 (s, 3H), 2.44 (s, 3H) ppm; **^13^C NMR (150 MHz, CDCl_3_)**: δ 140.85, 139.69, 135.95, 134.55, 130.76, 130.31, 127.50, 126.31, 125.91, 125.33, 21.29, 15.33.


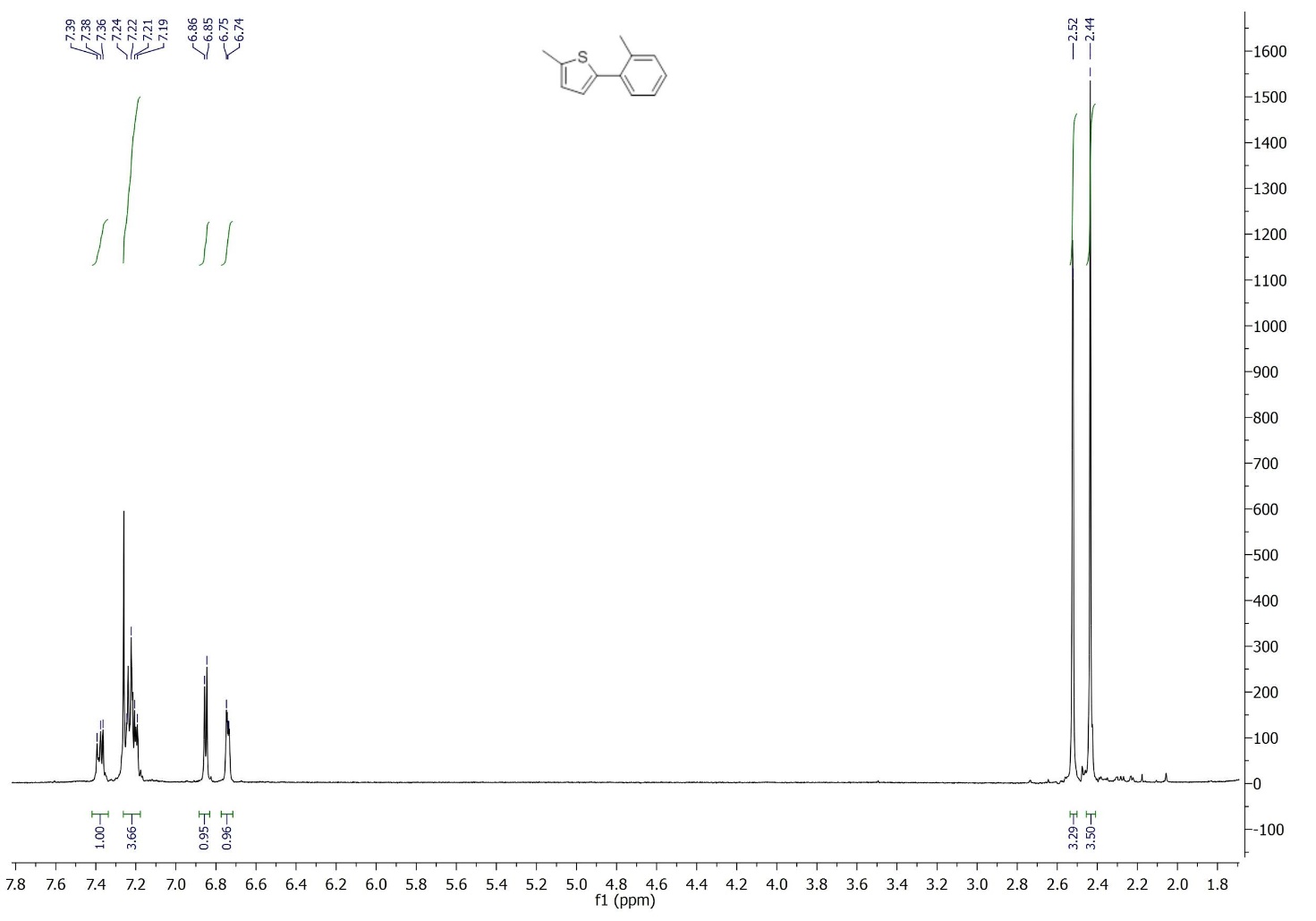


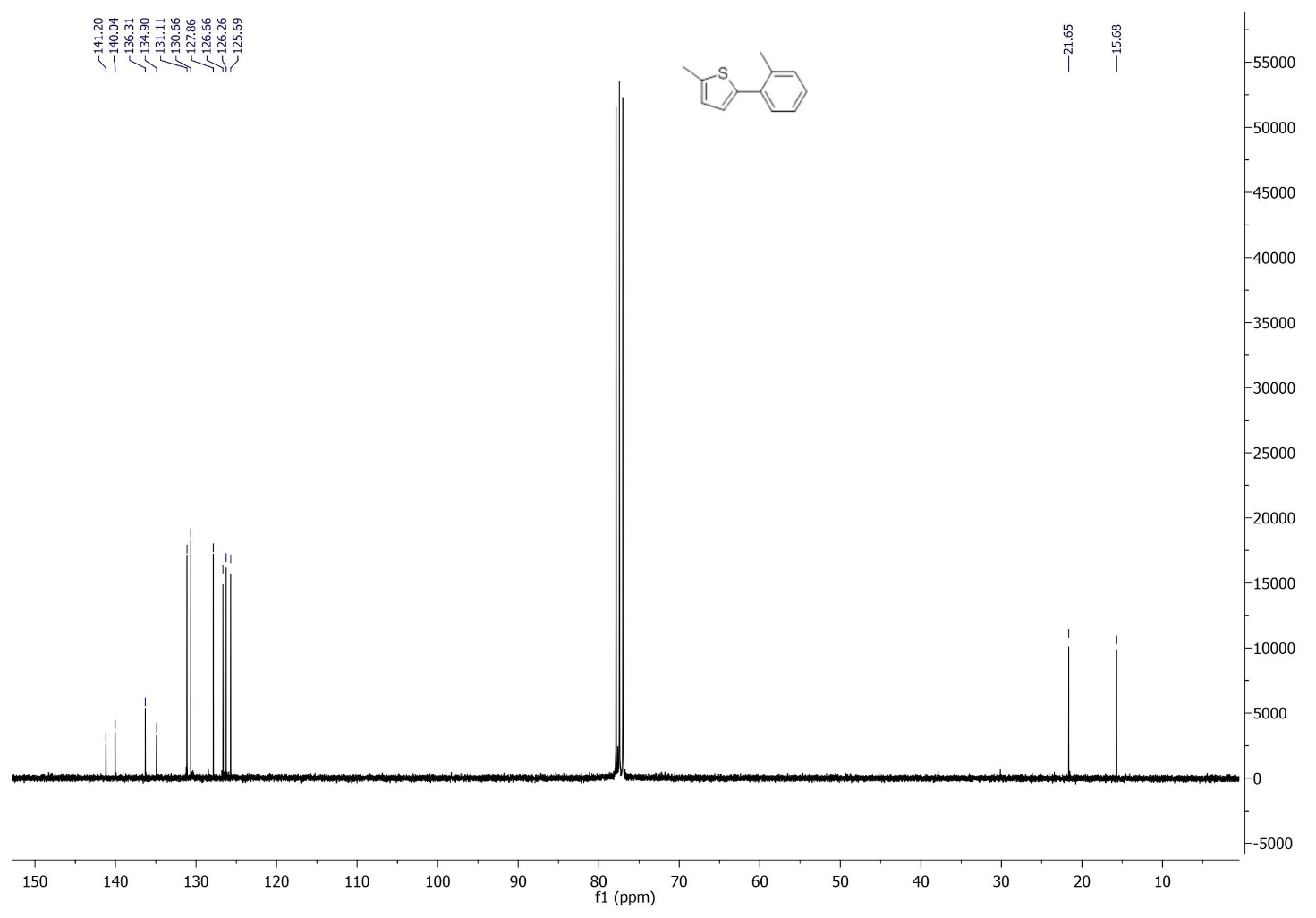


**3-(4-(5-methylthiophen-2-yl)phenyl)quinazolin-****4(3H)-one (6)**

**^1^H NMR (600 MHz, CDCl_3_)**: δ 8.38 (1H, ddq, J=7.9 Hz, 1.5 Hz, 0.5 Hz, H-9); 8.14 (1H, s, H-3); 7.81 (1H, t, J=7.0, H-8) e 7.77 (1H, dd, J=8 Hz, 0.4 Hz, H-6 ); 7.69 (2H, dt, J=8.7 Hz, 2.46 Hz, H-2’, H-11’); 7.55 (1H, t, J=7.02, H-7); 7.40(2H, dt, J=8.7 Hz, 2.46 Hz, H-3’, H-10’) 7.16 (1H, d, J=3.6, H-9’); 6.76 (1H, dq, J=3.6, H-8’); 2.52 (3H, d, J=0.96, H-7’) ppm; **^13^C NMR (150 MHz, CDCl_3_)**: δ 160.9, 147.9, 146.1, 140.9, 140.4, 135.9, 135.8, 134.7, 127.8, 127.7, 127.4, 127.3, 126.6, 126.5, 124.1, 122.4, 15.6


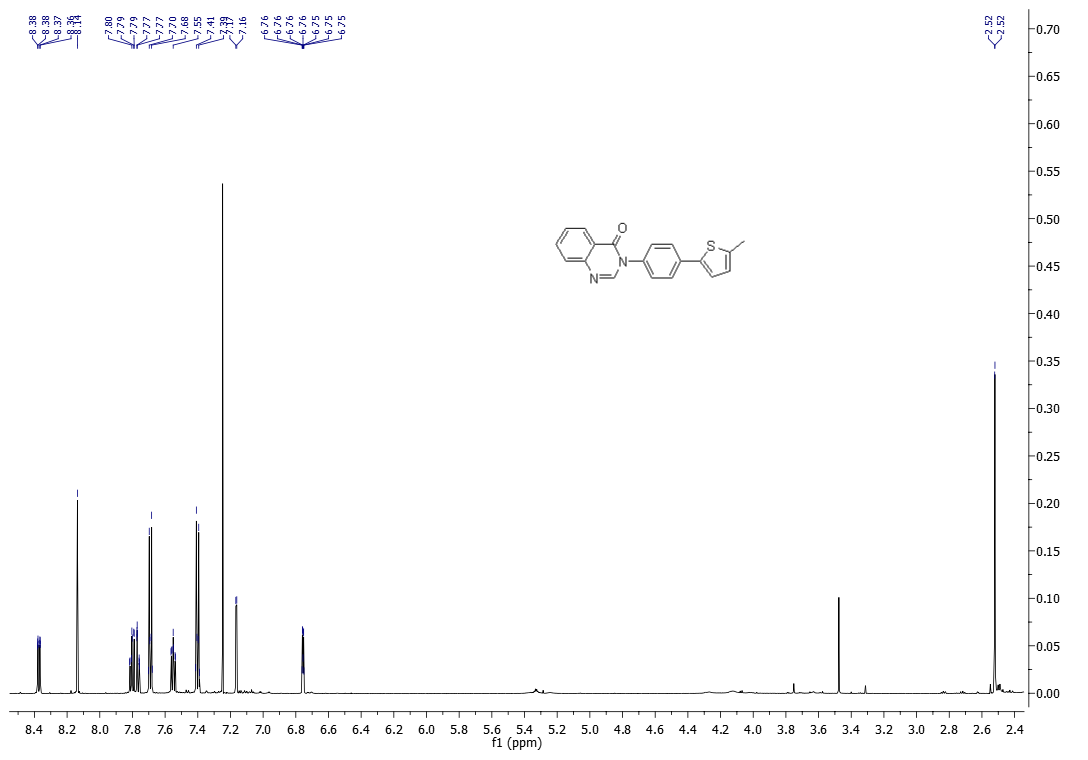


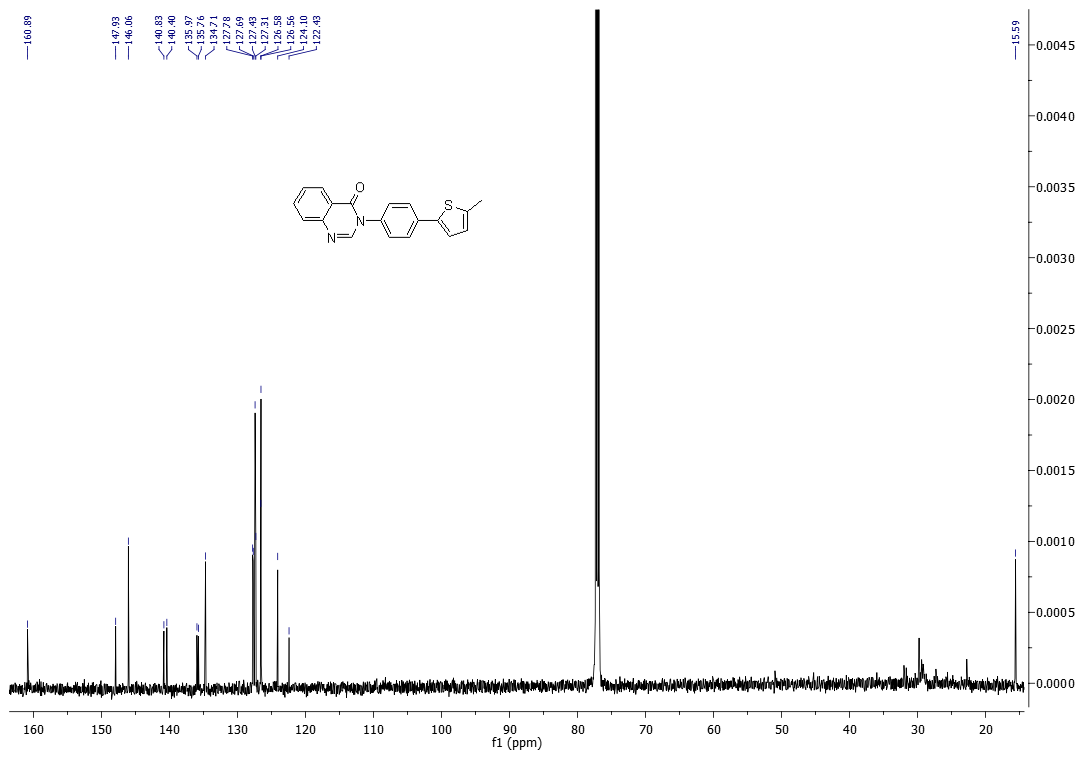

Supplement: Supplementary file 1 [file Data_Sheet_1.docx]
